# Novel motivational interviewing‐based intervention improves engagement in physical activity and readiness to change among adolescents with chronic pain

**DOI:** 10.1111/hex.14031

**Published:** 2024-03-31

**Authors:** Katalin Forgács‐Kristóf, Szilvia Ádám, Adrienn Vargay, János Major

**Affiliations:** ^1^ Doctoral School of Mental Health Sciences Semmelweis University Budapest Hungary; ^2^ Health Services Management Training Centre, Faculty of Health and Public Services Semmelweis University Budapest Hungary; ^3^ Institute of Psychology ELTE Eötvös Loránd University Budapest Hungary; ^4^ HRC Bethesda Children's Hospital Paediatric Pain Centre Budapest Hungary; ^5^ Institute of Behavioural Sciences Semmelweis University Budapest Hungary

**Keywords:** autonomy, motivational interviewing, paediatric chronic pain, physical activity, self‐management

## Abstract

**Introduction:**

Engaging adolescents with chronic pain in physical activities is challenging. Motivational interviewing (MI) combined with activity promotion may encourage teens to make behavioural changes. This research aimed to assess the feasibility and acceptability of our MI‐based physical activity promotion programme, the M3 training.

**Methods:**

In our exploratory study with 35 adolescent–parent dyads, we evaluated the feasibility by enrolment, drop‐out and retention rates. Acceptability of the M3 training was examined by adherence rates and participation experiences through open‐ended questions. We also assessed changes in pain self‐efficacy and readiness to change after the M3 training intervention.

**Results:**

The M3 training was feasible with an adequate enrolment (77.8%) and retention (85.7%) rate. Both teens and parents found the M3 training acceptable and considered exercise and physical activity the most helpful elements of the programme (36% and 37%, respectively). While self‐efficacy remained unchanged, we identified a significant increase in the readiness to change for adolescents and parents.

**Conclusion:**

M3 training improved physical activity engagement while prioritising adolescents' autonomy. Furthermore, it appears to be a clinically relevant approach and could result in a positive shift in readiness to change within a shorter timeframe.

**Patient or Public Contribution:**

The preliminary version of the M3 training was reviewed and commented upon by the public (adolescents and adults). Adolescents who participated in this study were designing their own movement programme, considering their lived experiences. Participants' feedback was used to create the online version of the M3 training (which will be published elsewhere).

## 1 INTRODUCTION

The embodied pain approach recognises that pain, as a bodily experience, can restrict current and future physical actions, emphasising that our ever‐changing bodies shape our experiences.^1^ Through action, we can alter our perception of the world.^1^ In chronic pain (CP), this exploration by action may be reduced, resulting in limited new bodily information and experiences.^2^ This is supported by empirical evidence showing that adolescents with CP have significantly lower daytime activity, more sedentary time and poorer subjective physical functioning than healthy adolescents.^3^ In contrast, aerobic and neuromuscular exercise may attenuate pain intensity for paediatric patients.^4^, ^5^ Exercise is a structured form of physical activity that involves repetitive movements to maintain or improve physical fitness.^6^ Hence, it is unsurprising that exercise promotion is a primary therapeutic objective when addressing CP. However, encouraging adolescents to exercise and maintain their active participation remains challenging.

Acknowledging the inherent variability between individuals is crucial, as several factors influence the final behavioural execution of activity endurance or avoidance. Multiple and sometimes conflicting goals may be concurrently present, potentially mitigating or exacerbating the relationship between pain‐related fear and avoidance behaviours.^7^, ^8^ For instance, physical activity greatly influences pain severity in some teens but not others.^9^ In addition, adolescents are more likely to approach activities when pain intensity is marked low. However, high‐pain‐intensity situations are more likely to be approached when the assigned goals are more important.^10^ Moreover, a recent study found that fear of pain in the afternoon predicted higher levels of evening activity avoidance. In contrast, greater positive affect and activity engagement in the afternoon predicted increased evening activity engagement among adolescents.^11^ Action and motivation are intrinsically intertwined, and to comprehensively examine the behavioural approach of adolescents towards activity avoidance‐endurance, incorporating a motivational perspective is strongly advised.^8^, ^10^

One recommended solution to engage adolescents in physical activities is motivational interviewing (MI).^12^ MI is designed to explore and resolve individuals' ambivalence about behaviour change and to increase the likelihood that they will make positive changes. As a patient‐centred approach, the spirit of MI involves partnership, acceptance, compassion and evocation and has four key processes: engaging, focusing, evoking and planning.^13^ Engaging is the process of establishing a strong working relationship with the client based on trust and respect. Through focusing, the direction of the conversation is set and influenced by the patient, resulting in a specific target behaviour to address. Evoking is a distinct feature of MI, where the patient's motivation for change is elicited through their arguments for change. During planning, the patient is encouraged to develop a change plan that is acceptable, reachable and suitable.^13^ To our knowledge, MI has never been investigated in the context of day‐hospital paediatric CP treatment to facilitate physical activity.

Due to the high workload and limited resources, there is an increasing need for brief, effective and accessible complementary interventions to address the high prevalence of CP among Hungarian teens.^14^ In the adult population with CP, brief intervention (BI) programmes have yielded promising results in reducing sick leave, overuse of medication, pain severity and catastrophizing, lowering barriers to pain management.^15^, ^16^, ^17^, ^18^ Thus, we developed a brief MI‐based intervention (M3 training) and implemented it to address this issue. The primary aim of our study was to describe the development of M3 training and to evaluate its feasibility and acceptance in the paediatric pain population. The secondary aim was to assess differences in adolescents' and parents' readiness to change and self‐efficacy pre‐ and posttraining. We hypothesised that the M3 training would be feasible and acceptable to adolescents in this context.

## 2 MATERIALS AND METHODS

### 2.1 Study design

The current study was a single‐arm, nonrandomized pilot feasibility study of a BI called M3 training, consisting of three sessions over a month. Data were collected from adolescents and their caregivers at baseline, before the first M3 training session, and after the third follow‐up session at 1 month. This study is part of an ongoing randomised controlled trial that was approved by the Hungarian National Ethical Committee (Approval number: IV/7741‐3/2021/EUK) and registered at clinicaltrials.gov (NCT05220384). All participants and their parents provided written consent.

### 2.2 Recruitment

**Figure 1:**
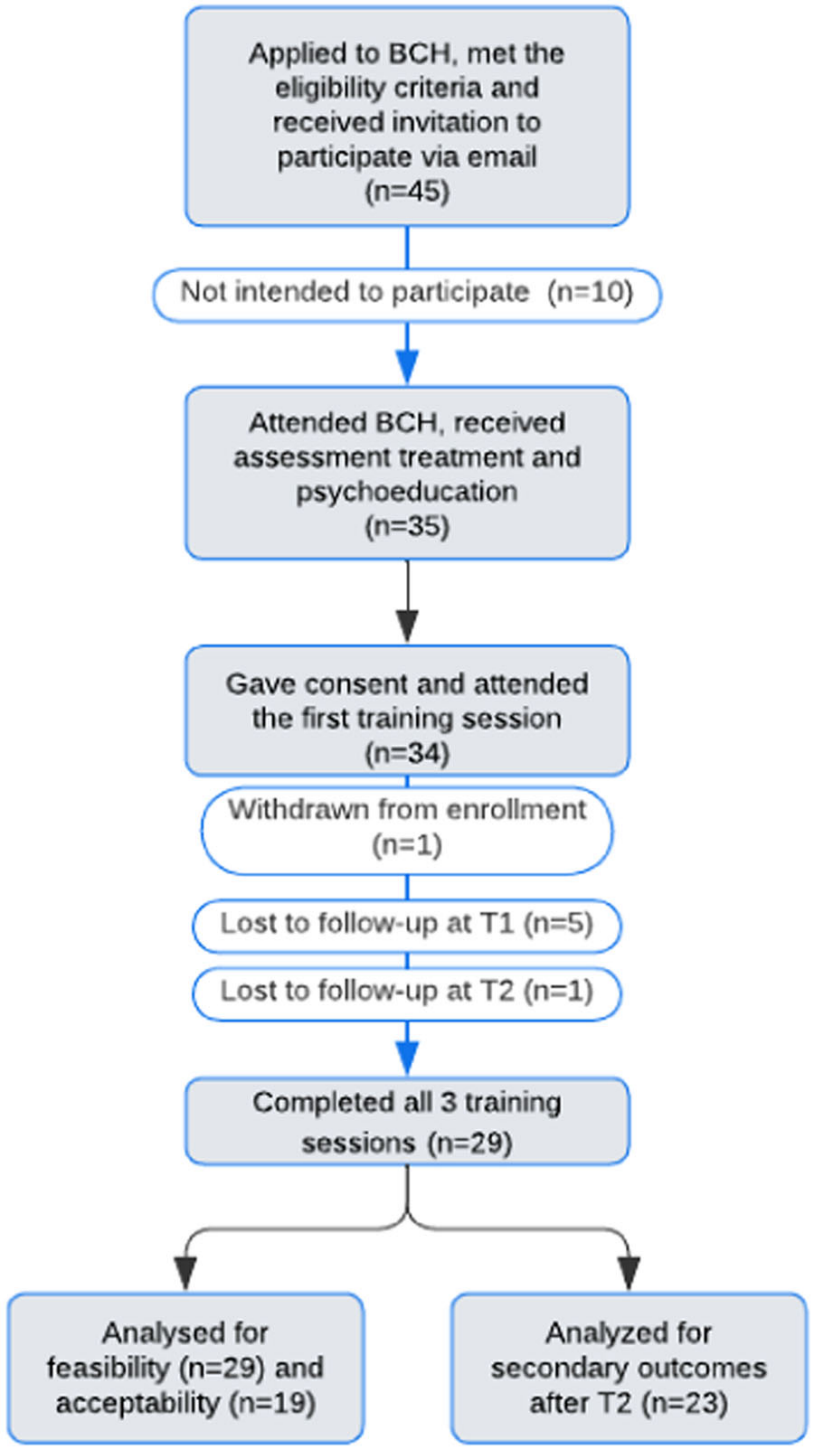
Flow diagram for the enrolment. BCH, Bethesda Children's Hospital; T1, 1‐week follow‐up; T2, 1‐month follow‐up.

Adolescents and their primary caregivers (*N* = 35) were recruited from the Paediatric Pain Centre (PPC) at HRC Bethesda Children's Hospital (BCH) from 1 February 2022 to 31 May 2023. BCH is the only interdisciplinary paediatric CP facility in Eastern Europe.^19^ Children and adolescents referred to this centre include those who experience various CP conditions, such as chronic headaches and abdominal, back, upper and lower limb pain. In the current study, individuals were eligible to participate if they (1) were between 12 and 18 years old; (2) had long‐lasting or recurring (at least three occurrences per week) pain for at least 3 months; (3) had no ongoing organic disease; (4) had good reading, writing and listening skills in the Hungarian language; (5) were capable of independent movement and cognitive reasoning and (6) had access to the internet and phone. Adolescents were ineligible to participate if they had (1) significant cognitive or physical impairment (e.g., cerebral palsy, infections) or (2) significant psychiatric problems that would interfere with the self‐management programme (e.g., psychosis). Figure 1 shows the flowchart of the study process.

### 2.3 Intervention development

Maintaining an authentic self and finding the right environment and activities imply that most choices and behaviours align with our inner desires and needs.^20^ The internal representations of our desired states are defined as goals.^21^ Examining the interaction of multiple competing goals is essential to understanding motivational processes. CP experience can hinder goal achievement, as individuals constantly remain vigilant, fear pain and engage in avoidance behaviours. Constant anticipation of pain can lead to forward planning, which may result in individuals perceiving certain activities as painful, even without concrete evidence, thereby affecting their engagement with these goals. We employed the MI approach to explore teens' ambivalences and facilitate their comprehension of associations between activities and pain experiences. MI in CP has long been recommended in adult and paediatric populations.^22^, ^23^ MI has been integrated into different approaches to managing adult CP, such as pain neuroscience education.^24^ There is growing evidence for using MI to improve psychosocial and physical health outcomes and facilitate behaviour change in youth.^25^ Given this consideration, the core components of our novel programme were motivation, movement and self‐management, hence the name M3 training. According to the autonomy‐control continuum incorporated into Self‐Determination Theory, autonomously motivated behaviours are experienced as volition and an expression of one's self.^26^ Choosing movement and activity as the frame of the M3 training, we created a boundary wherein adolescents can explore their desires and seek their autonomous motivation without feeling compelled to act in a previously determined way. The brief M3 training intervention was designed by four CP experts in different fields. The team comprised a CP physiotherapist (PT) (K. F‐K.), a paediatric CP medicine specialist (J. M.), a mental health management expert (SZ. Á.) and a paediatric clinical psychologist (A. V.). The process contained four stages: (1) literature review, (2) setting up the MI programme, (3) framing the M3 training and (4) designing the workshop.

#### 2.3.1 Literature review

We conducted a literature review on PubMed, Web of Science and Scopus. The following keywords in the title and abstract were used: ‘motivational interviewing’ AND (‘motivation*’ AND ‘chronic pain’ AND [‘exercise’ OR ‘physical activity’] AND [‘pain management’ OR ‘behaviour change’]). Reference lists of the relevant papers were also scanned. Moreover, we reviewed the following three books, as they explicitly discuss pain, motivation and motivational interviewing: ‘Motivational Interviewing helping people change’,^13^ ‘Motivational Perspectives on Chronic Pain’ with specific attention on Chapters 6, 8, 9 and 11,^27^ and ‘Embodied: the psychology of physical sensation’ with particular attention on Chapter 7.^28^

#### 2.3.2 Setting up the MI programme

Based on the literature review results, several possible open‐ended questions were created regarding adolescents' pain experience, activity, movement‐related goals, perceived obstacles and motivators for exercising. We collected these questions in a document and drafted the initial structure of the BI.

#### 2.3.3 Framing the M3 training

**Figure 2:**
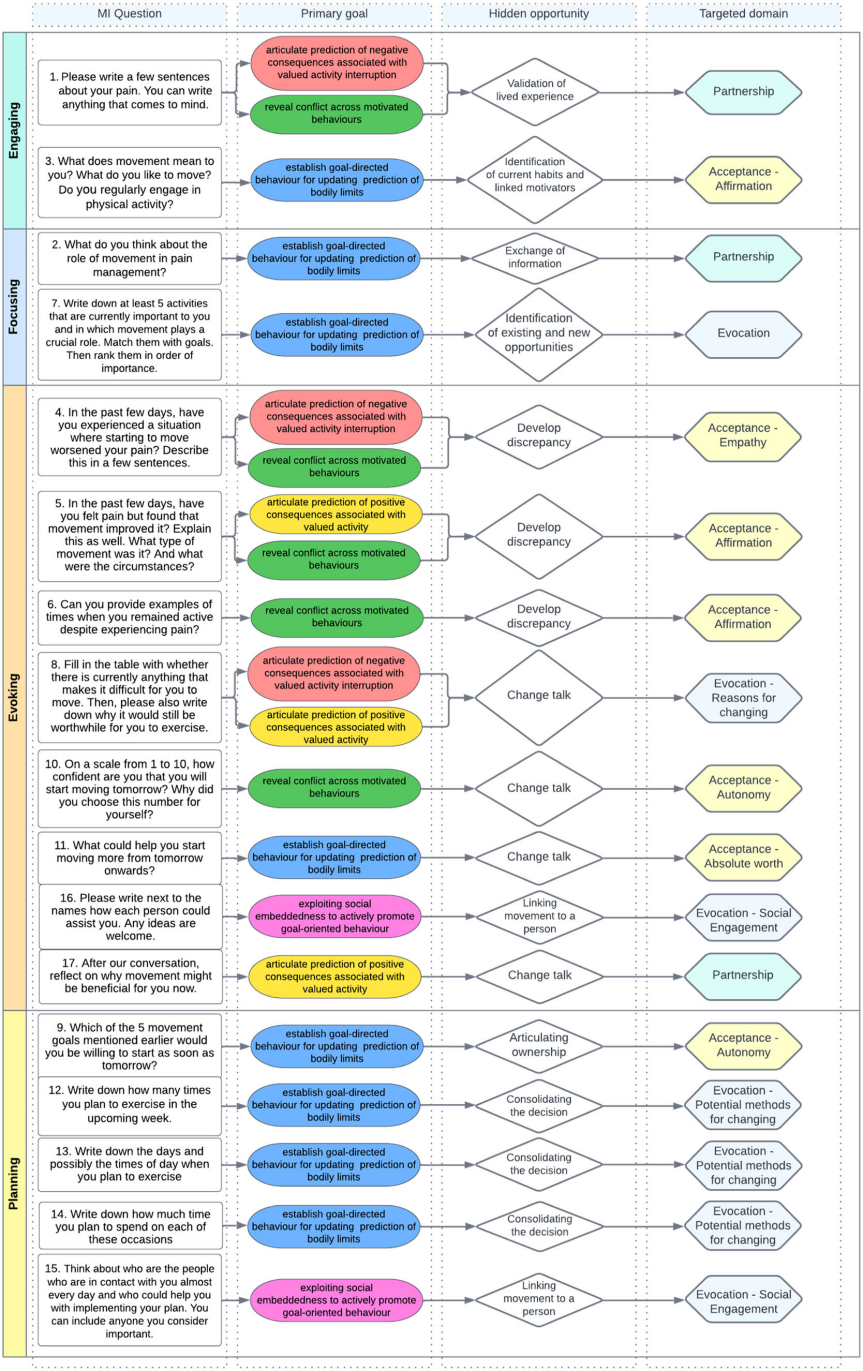
The final form and the theoretical background of M3 training. All questions were considered and structured to cover the four stages of MI. Each inquiry has a ‘Primary goal’ built upon the embodied pain: clinical agenda proposed by Tabor et al.^2^ These were linked with ‘Hidden opportunities’ that will likely emerge during the M3 training, allowing the therapist to utilise them to facilitate behavioural change. Finally, ‘targeted domains’ reflect the spirit of MI, ensuring the collaborative and patient‐centred nature of the M3 training. MI, motivational interviewing.

Consensus on the inclusion of the specific MI questions was sought over three rounds of online consultation, where the four members of the multidisciplinary expert team gave their feedback on the content. Five adolescents (one teen who experienced CP in the past and four healthy youth) and five adults (three medical students, one parent of adolescents and one adult who lives with CP) were also asked for their feedback on the preliminary structure and tone of the M3 training questions. We aimed to ensure age‐appropriate language with clear and easily understandable terms. We selected the final MI questions based on the collective feedback. Some questions were rephrased to guarantee positive suggestions to maximise motivation, inspire, boost confidence and facilitate behaviour change.^29^ For instance, we changed the order in Question 8, asking first about the difficulties and then the advantages. In Question 12, we changed the wording from ‘…how many times you would move…’ to ‘how many times you plan to exercise’. MI is a scientifically supported intervention that relies on the fundamental concepts of patient‐centred counselling and adopts a collaborative and nonconfrontational strategy.^30^ It further acknowledges the individual's varying involvement in a particular behaviour. The concept of readiness to change behaviour stems from the transtheoretical model of behaviour change, which proposes that an individual's readiness to engage in behaviour change is a fundamental prerequisite for actual change. This theoretical framework identifies five stages of change: precontemplation, contemplation, preparation, action and maintenance.^31^ In designing our training programme, we considered these stages and aligned them with the four stages of MI. Hence, the M3 training structure was designed to have questions addressing each stage, giving the practitioner the opportunity and flexibility to progress accordingly during the training. The final structure followed a logical line from broader, more abstract questions (i.e., ‘What is the first thing that comes to your mind about pain?’ ‘What does moving mean to you?’) to the more concrete ones (i.e., ‘What is the activity you are willing to start tomorrow?’, ‘On which day will you do it?’). These questions formed the main components of M3 training and were delivered verbally. Figure 2 shows the final questions with the goals and theories they tend to represent.

#### 2.3.4 Designing the workbook

The structured and grouped MI questions were printed out as a workbook. A graphic designer created the visual and design of the materials. The final design of the workbook can be found in Appendix S1.

### 2.4 Measures

#### 2.4.1 Primary outcomes

The primary outcomes of this study focused on the feasibility and acceptability of the M3 training. Following Orsmond et al.'s^32^ recommendations, feasibility was evaluated using enrolment rates, drop‐out rates, retention rates and reasons and engagement with the training. Acceptability was measured through adherence rates and a structured, open‐ended question (‘What did you find most useful from the programme; what has helped the most?’) at the end of each session.^33^ Adolescents and their caregivers were invited to answer a written question at the end of the trial: ‘What do you think about the M3 training programme?’. Our goal was to evaluate the participant's perception of the intervention and determine the most beneficial aspect of it.

#### 2.4.2 Secondary outcomes

Secondary outcomes to evaluate the impact of M3 training included pain‐related domains (e.g., pain self‐efficacy and readiness to change). The specific measures listed below were translated into Hungarian, following the recommended forward–backward process.^34^

##### Baseline characteristics

A Hungarian version of the German Pain Questionnaire for children, adolescents and parents^35^ was administered at baseline to gather demographic and health information, such as sex, age, pain diagnosis, pain sites, duration of pain and presence of comorbidities. The 11‐point numeric rating scale assessed the maximum and average pain intensity over the previous week.^36^

##### Pain self‐efficacy

The Pain Self‐Efficacy Scale Child and Parent versions (PSES‐C, PSES‐P) were used to measure self‐efficacy concerning normal functioning when in pain.^37^ Example items from the child version include ‘How sure are you that you can take care of yourself when you have pain?’ and from the parent version, ‘How sure are you that your child can do well in school when in pain?’. Higher scores on a five‐point Likert scale indicate poorer self‐efficacy on both scales. Initial validation supported the measure's validity and reliability.^37^ In the current sample, the alpha coefficients of the adolescent and parent data were *α* = .743 and *α* = .906, respectively.

##### Readiness to change

The Pain Stages of Change Questionnaire for Adolescents and for Parents (PSOCQ‐A, PSOCQ‐P) was used to measure adolescent and parent motivation to change and readiness to adopt a self‐management approach to CP.^38^ The 30‐item parallel measures were rated on a five‐point Likert scale, ranging from 1 (Strongly Disagree) to 5 (Strongly Agree), with higher subscale scores representing the current dominant stage of change. In the English version, the distinct domains for parents' readiness are pre‐contemplation, contemplation, action and maintenance, while for youth, a three‐factor structure was found combining action/maintenance as one.^38^ The Cronbach's *α* for the teen version was *α* = .789, and that for the parent version was *α* = .772.

### 2.5 Intervention

#### 2.5.1 Standard interdisciplinary pain treatment (SIPT)

The PPC at BCH in Hungary has provided SIPT for those children and their families who have sought help to manage CP since 2013. SIPT is a 2‐h‐long, in‐person intervention delivered by a physician (paediatrician or anesthesiologist) and a clinical psychologist specialising in paediatric CP. The whole family is invited to participate. The pain specialist provides a physical examination after covering the biological, psychological and social contributors to the pain problem. After the assessment, the two healthcare professionals provide psychoeducation and discuss personalised behaviour change strategies, such as sleep habits, distraction methods, stress management, meaningful hobbies and relationships, eating and drinking habits, medication use (including misuse and overuse) and psychotherapy options, if necessary. The family is expected to return for a 3‐month follow‐up session to discuss any changes during this period. The M3 training is an add‐on opportunity for families with teenagers over 12 years old to explore exercise as a possible pain management solution.

#### 2.5.2 M3 training

The M3 training was led by a PT with a master's degree, a postgraduate degree in CP treatment and training in MI. The first individual in‐person session started after the SIPT. The discussion occurred in a quiet room, where the youth and one of their caregivers were asked to provide written consent to participate and complete the patient and parent questionnaires (i.e., PSES, PSOCQ). The therapist gave an overview of what to expect during the first session and then asked the youth the M3 training questions. The attending parent was invited to share their thoughts and opinions on the topic of discussion. PT also encouraged the participants to express their genuine thoughts and feelings concerning their pain, movement and how the two interrelate. After discussing each query, the teen jotted down their responses in the workbook (see Figure 2 for questions and Appendix S1 for the workbook.) The PT gave a summary after each conceptual block. Open‐ended questions were used to progress the discussion and affirmation and empathy were expressed frequently during the session. The final goal of the training was to create a movement plan in collaboration with the adolescent that considers their autonomy, necessities, pain‐related behaviours and reasoning. The PT did not force any activity and allowed adolescents to express their ideas regarding what, when and how to exercise. Each exercise plan was unique, varying in frequency and intensity (to get an overview of the selected exercise plans, please refer to Appendix S2.) The focus often shifted from activity planning to activity optimisation with the integration of progressive relaxation and diaphragm breathing techniques. The parents frequently played a significant role in implementing the exercise plan. Adolescents typically asked their parents to remind them, gently persuade them and sometimes participate in their chosen activities. All participants were provided supplementary materials after the first training appointment, such as audio‐recorded breathing exercises and progressive relaxation, and a written version of these. Appendix S3 shows the written version of the materials. In cases where adolescents needed more guidance regarding exercises, an online application (PhysiApp) was used to create the desired movement plan.

The second consultation was a phone follow‐up session after 7–10 days (T1). The PT sent a text message to the parent 1 day before the scheduled phone call, requesting confirmation. During the call, the adolescent and parent reflected on their week regarding the exercise plan and pain. Youth expressed their successes or failed tries and articulated how they intended to move forward or overcome the barriers they faced during the first week. The parent shared their view on their child's progress and how they helped implement the exercise plan. If necessary, changes to the plan were made. The third phone session took place 28–30 days (T2) after the previous call, following the same structure. The follow‐up questionnaires (i.e., PSES and PSOCQ) were sent via e‐mail after each phone consultation.

### 2.6 Statistical analysis

Data analyses were performed using SPSS version 29.0 (IBM® SPPS® Statistics; IBM Corp). Feasibility and satisfaction were evaluated using descriptive statistics (means, standard deviations, frequencies). Means and standard deviations were calculated for the secondary outcome measures at preintervention (baseline) and 1‐month follow‐up (T2). The baseline questionnaires were paper‐based, and as a result, some data were missing. Those cases where missing data was higher than 10% were eliminated from the data set. The remaining data were missing completely at random. Missing data of incomplete baseline readiness variables were calculated with multiple imputation models with 10 iterations using predictive mean matching.^39^ The follow‐up questionnaires were electronically registered. We screened the data for normality by inspecting the values of skewness and kurtosis.^40^ Intervention effects were explored using paired *t*‐tests to assess the outcome changes between the two‐time points. Cohen's *d* effect sizes were also calculated, with effect sizes of 0.2, 0.5 and 0.8 considered small, medium and large effects, respectively.^41^ Responses to the open‐ended questions in the follow‐up survey and initial intervention session were reviewed and thematically grouped to identify valuable elements of the intervention.

## 3 RESULTS

### 3.1 Participants' characteristics and changes in activity regularity

Adolescents who participated were predominantly female (*n* = 35, 68.6%), with a mean age of 15.26 (SD ± 1.89) years. Headache (tension‐type, migraine, mixed type) was the most frequent primary pain type (*n* = 16, 45.7%), and abdominal pain was the second most common (*n* = 7, 20.0%). Over half of the adolescents had multiple painful sites (*n* = 21, 60.0%). The demographic characteristics of the youth are summarised in Table 1.

**Table 1:** Characteristics of participants.

Characteristics	Participants (*n* = 35)
Age (mean [SD])	15.26 (1.89)
Gender	
Male (%)	11 (31.4)
Female (%)	24 (68.6)
Painful region^a^	
Head (%)	16 (45.7)
Neck (%)	1 (2.9)
Lower back (%)	3 (8.6)
Upper extremity (%)	2 (5.7)
Lower extremity (%)	6 (17.1)
Abdominal (%)	7 (20.0)
Multiple regions (%)	21 (60.0)
Duration of pain	
3–6 months (%)	3 (8.6)
6–12 months (%)	5 (14.3)
1–2 years (%)	9 (25.7)
2–5 years (%)	7 (20.0)
>5 years (%)	11 (31.4)
Pain intensity^b^ (mean [SD])	
Average	4.9 (2.0)
Maximum	6.9 (2.3)
Family structure	
Nuclear (%)	27 (77.1)
Divorced parents (%)	8 (22.9)
Missed school days in the last 3 months (mean [SD])	12.86 (21.19)
Regular sport in the past	
Yes (%)	27 (77.1)
Competitive (%)	10 (37.0)
No (%)	8 (22.9)
Regular sport now	
Yes (%)	12 (34.3)
Competitive (%)	3 (25.0)
No (%)	23 (65.7)
Comorbidities	
1 Diagnosis (%)	2 (5.7)
2 Diagnoses (%)	7 (20.0)
3 Diagnoses (%)	11 (31.4)
3+ Diagnoses (%)	15 (42.9)

^a^ The most dominant painful site.

^b^ Numeric rating scale (NRS: 0−10).

### 3.2 Feasibility of study recruitment and enrolment

Over 15 months, 45 families who met the eligibility criteria were invited to participate in the study. Of those, 35 (77.8%) expressed interest, completed the study questionnaires and started the training. One family did not wish to pursue further after the first M3 training session. Of those who enroled in the M3 training, 29 adolescent–parent dyads (85.7%) completed the intervention. Five families dropped out at the first follow‐up, and one additional family dropped out at the second follow‐up. The reasons for the termination of the M3 training included failed responses to text reminders by parents (*n* = 4) and family emergencies (*n* = 2). The mean length of the first in‐person session was 78 min (SD = 21.2), while the mean lengths of the second and third consultations were 19 min (SD = 7.4) and 15 min (SD = 9.8), respectively. The average number of days between the first and second consultation was 9.5 days (SD = 5.5), while between the second and third phone sessions, it was 22.5 days (SD = 9.5).

### 3.3 Acceptability

During the second phone consultation, 29 adolescents (82.9%) reported that they started implementing their preplanned activity programme. Of those, 17 youth (48.6%) fully and eight (22.9%) partially accomplished their chosen activities by the second follow‐up consultation. Adolescents' and parents' response rates to follow‐up questionnaires at T2 were 62.8% and 65.7%, respectively.

The participating families were invited to answer the question: ‘What did you find most useful from the programme?’. Nineteen youth (54.3%) and 15 parents (42.8%) responded. Four themes emerged after thematic appraisal: exercise and activity, attitude change, attention distraction and relaxation, motivation and continuous feedback (Table 2). Both teens and parents considered exercise and activity (36% and 37%, respectively) to be the most valuable elements of the programme. The second most frequently considered elements were attention distraction, motivation and feedback.

**Table 2:** Feedback quotes and themes from adolescents and parents.

Prompt	Theme	Illustrative quotes
What has been the most useful for you in the programme? What has helped you the most?	Exercise and activity	‘More movement. That helped the most, I think’. (Teen) ‘The fact that I could start moving again’. (Teen) ‘The encouragement of physical activity’. (Parent)
	Motivation and feedback	‘The fact that they control my movement and that motivates me to do it. And to be able to report my successes to others afterwards’. (Teen) ‘The program itself, including the physiotherapist motivational talk’. (Parent)
	Attention distraction and relaxation	‘I tried not to think about it, and it made my leg hurt less’. (Teen) ‘Distraction helped more with anxiety, and relaxation helped with sleep’. (Parent)
	Attitude change	‘I stopped taking the pill because I realized it was no good’. (Teen) ‘Regularity, consistency’. (Parent)
What do you think about the M3 training?	‘I didn't feel that I was being forced here and that I was being put down because of my previous decisions and actions. It felt more like an encouragement, where we talked through and found a solution to a problem together. It felt good that there was no instruction or scolding and that you sought to understand me’. (Teen)	
	‘The best thing, I think, is that it is achievable. It was very interesting to see that he was not allowed to move for a few days because of the dental treatment, and when the mandatory rest period was over, he asked if he could move and went up to his room and did what he had to do (referring here to the exercises)!’ (Parent)	
	‘This movement has worked. Because really, if I've had a really bad day, but I go to the gym and I get out and about, then I have a sense of relief afterwards, or that at least I've done this. It gives me a sense of pride that I did it. And it really feels good afterwards to walk until I get home and feel more liberated. And I sleep better on those days’. (Teen)	
	‘Perhaps the most important thing was, first of all, that he felt that he could do for himself and that what he could do for himself was not beyond his mood and his abilities, and that motivated him to do this M3 program. Perhaps the most important thing is that he wanted to do it’. (Parent)	

### 3.4 Self‐efficacy and pain stages of change

**Figure 3:**
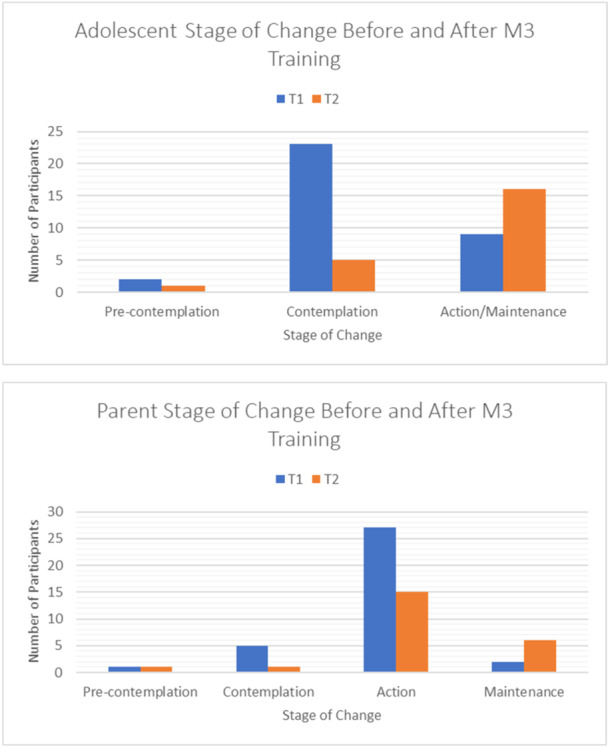
Adolescent and parent readiness to self‐manage pain before and after M3 training.

At baseline (T1), based on the PSOCQ scores, 5.9% of adolescents were classified in the precontemplation stage, 67.6% were classified in the contemplation stage and 26.5% of youth were classified in the action/maintenance stage. After the 1‐month follow‐up of the M3 training (T2), 4.5% of adolescents were classified in precontemplation, 22.7% in contemplation and 72.2% in action/maintenance. Parent scores showed a similar shift towards the maintenance stage. For a summary of the stage changes (see Figure 3).

For adolescents, results of paired t‐tests comparing T1 and T2 scores in readiness ratings showed significant changes. Baseline precontemplation scores (*M* = 2.27, SE = 0.17) decreased compared to 1‐month follow‐up scores (*M* = 2.03, SE = 0.15). This difference was significant *t*(21) = −2.08, *p*= .05, and it represented a small‐sized effect *d* = −0.44. There was a change in the action‐maintenance scores between T1 (*M* = 3.49, SE = 0.16) and T2 (*M* = 3.95, SE= 0.15). This increase was significant *t*(21) = 3.03, *p* = .006 and indicated medium effect size *d* = 0.65. Regarding parental readiness ratings, there was a significant increase in action between T1 (*M* = 4.40, SE = 0.10) and T2 (M = 4.59, SE = 0.11, *t*(22) = 2.62, *p* = .016), representing a medium effect size *d* = 0.55. Parents' maintenance scores between T1 (*M* = 2.78, SE = 0.23) and T2 (*M* = 4.17, SE = 0.12) also significantly increased *t*(22) = 5.15, *p* < .001, and this represented a large effect size *d* = 1.07. However, self‐efficacy for both youth and parents remained unchanged throughout the first month. For a summary of the secondary analysis, see Table 3.

**Table 3:** Secondary analysis of pre and posttreatment differences of stages of change and self‐efficacy.

Variables	Baseline		1‐month follow up		Mean difference (95% CI)	*p*‐Value	*d*‐Value
	*N* (%)	mean (SD)	*N* (%)	mean (SD)			
Self‐efficacy (adolescent)^a^	35	3.17 (0.66)	22	2.83 (0.95)	−0.03 [−0.44, 0.38]	.895	−0.214
Self‐efficacy (parent)^b^	35	2.82 (0.93)	23	2.56 (1.11)	−0.22 [−0.67, 0.23]	.316	0.159
Stages of change (adolescent)^c^	34		22				
Precontemplation	2 (5.9)	2.45 (0.75)	1 (4.5)	2.03 (0.71)	−0.23 [−0.46, −0.00]	**.050** *	−0.444
Contemplation	23 (67.6)	3.52 (0.64)	5 (22.7)	3.60 (0.72)	0.06 [−0.16, 0.29]	.570	0.123
Action/maintenance	9 (26.5)	3.33 (0.76)	16 (72.2)	3.95 (0.70)	0.46 [0.15, 0.78]	**.006** *	0.646
Stages of change (parent)^d^	35		23				
Precontemplation	1 (2.9)	2.31 (0.82)	1 (4.3)	2.05 (0.89)	−0.15 [−0.38, 0.08]	.196	−0.278
Contemplation	5 (14.3)	3.92 (0.64)	1 (4.3)	3.85 (0.93)	−0.16 [−0.45, 0.14]	.279	−0.231
Action	27 (77.1)	4.47 (0.49)	15 (65.2)	4.59 (0.54)	0.19 [0.07, 0.04]	**.016** *	0.545
Maintenance	2 (5.7)	2.84 (1.11)	6 (26.1)	4.17 (0.59)	1.39 [0.27, 0.83]	**.000** *	1.073

Abbreviation: CI, confidence interval.

^a^ Pain Self‐Efficacy Scale Child version and Parent version (PSES‐C).

^b^ Pain Self‐Efficacy Scale Parent version (PSES‐P).

^c^ Pain Stages of Change Questionnaire for Adolescents (PSOCQ‐A).

^d^ Pain Stages of Change Questionnaire for Parents (PSOCQ‐P).

* *p* ≤ .05; *d* = Cohen's *d*.

## 4 DISCUSSION AND CONCLUSION

### 4.1 Discussion

The objectives of this study were to demonstrate the developing processes and pilot testing of a novel MI‐based activity promotion intervention, the M3 training, specifically designed for adolescents with CP to improve activity and self‐management engagement. We also investigated the feasibility and acceptance of the M3 training. By analysing the data collected from a sample of 35 adolescents and their parents, we also aimed to provide valuable insights into the effectiveness of these interventions on readiness to change and pain self‐efficacy.

Our results indicate that the M3 training programme exhibits potential feasibility and acceptability among adolescents living with CP, which aligns with a recent study in which a novel telehealth MI‐based intervention (PREPaRe) to enhance readiness to change before engaging in intensive interdisciplinary pain treatment (IIPT) was also found to be feasible and acceptable to families of youth with persistent pain.^42^ However, this programme did not address movement and exercise as the primary intervention component.

We observed a significant shift in the action and maintenance stages of readiness to change, both among teens and parents, 1 month after the first M3 training intervention. Previous research has indicated that IIPT programmes, spanning 3–4 weeks, have improved children's readiness for self‐managing pain.^43^ In contrast, the M3 training programme achieved comparable outcomes with a significantly shorter intervention consisting of only 2 h of consultation. This could be attributed to several factors. First, adolescents' involvement in the decision‐making process regarding pain management strategies may contribute to a sense of empowerment, fostering feelings of competence and engagement.^44^ This participatory approach could shift their readiness from a contemplative state to one of action and maintenance. Consequently, adolescents may be more inclined to actively participate in these activities. Second, the malleability of readiness to change as a construct should be considered. Studies on adults with dependencies have shown that change talk and change plan are essential elements of MI that may facilitate readiness progression.^45^, ^46^ During the M3 training, open‐ended questions and summaries were utilised to elicit change talk^47^ and ambivalence. This may influence readiness to change and facilitate positive outcomes within a shorter timeframe.

The M3 training was well received by the participants, with a high participation rate regarding the follow‐up calls, and most of them successfully achieved their movement‐related goals. Among the respondents who provided feedback on the programme's utility, exercise and increased physical activity were frequently cited as the most beneficial aspects, followed by motivation and feedback. Holt et al.^48^ review revealed that long‐term adherence to musculoskeletal therapy in children is hindered by factors, such as lack of motivation, therapy monotony and pain during treatment. Despite the limited 1‐month duration of our study, empowering adolescents to choose and perform exercises autonomously showed promise in sustaining their engagement and adherence. This autonomy may encourage the development of new behaviours, leading to different bodily experiences and influencing their pain perception.

The Goal Pursuit Model for paediatric CP conceptualises the interplay of a child's motivation and fear of pain, as well as parent expectations and anxiety in pursuing goal achievement, resulting in approaching or avoiding activities. This model also proposed that targeting children's self‐efficacy and motivation could increase the likelihood of actively pursuing a goal.^49^ In line with this framework, one of the primary goals of the M3 training programme was to enhance motivation to move and self‐efficacy among youth despite pain. Our results showed no difference in self‐efficacy over 1 month. This lack of change could be attributed to the possibility that self‐efficacy beliefs may be more resistant to change for adolescents who live with CP.^50^ An individual's perception of their ability to perform a set of specific behaviours is a deeply ingrained concept and varies in level, strength and generativity across different domains.^51^ While readiness to change appears to be a more adaptable construct, self‐efficacy may represent an important target for future treatment approaches and research efforts. This may be even more complex in adolescents, considering that parents' self‐efficacy beliefs, past experiences and expressed support^37^ are influencing factors. These aspects may not have been addressed during the M3 training. Further research is required to specify the time length and the individual characteristics of youth for whom an intervention such as M3 training effectively augments self‐efficacy.

Despite the valuable insights, several limitations should be acknowledged. The small sample size and the self‐reported scores may be subject to biases; therefore, caution is advised when interpreting secondary outcomes. Although most participants completed the study, many individuals did not respond to the outcome questionnaires at the T2 timepoint. This indicates a significant drop‐out rate that could be due to redundancy and the time it took to complete the questionnaires. A more efficient data collecting system (i.e., automated daily reminders or sending questionnaires in smaller units) might solve these problems in the future. It is also important to consider that some participants may have ceased attending due to reasons that have not been explored. To better assist them in the future, it is necessary to delve deeper into adolescents' individual characteristics and environment, as they may require a more complex therapy. However, for those who are willing to take control and are ready to make changes with guidance, BIs such as M3 training may prove effective. Two‐thirds of the participants were female, which limits our understanding of the intervention's effects on males. Moreover, the study had no control group, and the follow‐up was short‐term. Furthermore, the same person conducted the interviews and analysed the data, which may impact the objectivity and validity of the study's findings. Finally, the questionnaires in the study were not fully validated in Hungarian; however, preliminary factor analyses confirm the factor structure of the original questionnaires (data not shown). To address these issues, we are administering a prospective randomised controlled trial with a 3‐month follow‐up to investigate the effectiveness of M3 training further.

### 4.2 Conclusion

In conclusion, our study provides evidence supporting the feasibility and acceptance of M3 training in motivating adolescents to engage in physical activity and changing their readiness for pain self‐management. We observed a high rate of adolescent activity involvement and positive feedback on the usefulness of activity in managing their pain. The significant shift in readiness to self‐manage pain implies that healthcare professionals should consider implementing interventions of movement promotion combined with MI, such as the M3 training, as part of their treatment strategies for teens living with CP. These interventions, involving a combination of motivation, movement and self‐management, can provide a holistic approach to pain management and improve patient outcomes. Moreover, it is time‐efficient and possibly cost‐effective, essential to all new therapies.

## AUTHOR CONTRIBUTIONS

**Katalin Forgács‐Kristóf**: Conceptualisation; methodology; investigation; writing—original draft; writing—review and editing; visualisation; formal analysis; project administration; funding acquisition; data curation; software; resources. **Szilvia Ádám**: Supervision; writing—original draft; writing—review and editing; conceptualisation; methodology. **Adrienn Vargay**: Conceptualisation; methodology; writing—original draft; writing—review and editing. **János Major**: Conceptualisation; writing—original draft; writing—review and editing; methodology; supervision; resources.

## CONFLICT OF INTEREST STATEMENT

The authors declare no conflicts of interest.

## Supporting information


**Appendix 1**. M3 Training Workbook.

**Appendix 2**. CERT checklist and individual exercise programs.

Appendix 2.1 Individual exercise program (A5).

Appendix 2.2 Individual exercise program (A8).

Appendix 2.3 Individual exercise program (A13).

Appendix 2.4 Individual exercise program (A17).

Appendix 2.5 Individual exercise program (A28).

Appendix 2.6 Individual exercise program (A32).

Appendix 2.7 Individual exercise program (A33).

**Appendix 3**. Progressive muscle relaxation and breathing exercise scripts.

## Data Availability

The data that support the findings of this study are available from the corresponding author upon reasonable request.
