# Supplementary material for: Novel motivational interviewing‐based intervention improves engagement in physical activity and readiness to change among adolescents with chronic pain
Source: Health Expect. 2024 Mar 31;27(2):e14031. doi: 10.1111/hex.14031 (PMC10982597; doi:10.1111/hex.14031)
Supplement: Supplementary file 1 — Appendix 1. M3 Training Workbook. [file HEX-27-e14031-s005.pdf]

M³ TRÉNING

Motiváció, Mozgás, Ön-Menedzsment Tréning

Önmenedzselő Munkalap

Szia! Örülök, hogy úgy döntöttél, hogy részt veszel a Bethesda Kórház Fájdalomkezelő Ambulancia és a Semmelweis Egyetem közös programjában. Ez az Önmenedzselő Munkalap abban lesz a segítségedre, hogy megtaláljuk a mozgással kapcsolatos céljaidat és megtervezzük, hogy hogyan tudod azokat elérni.

A program célja az, hogy jobban megismerd a fájdalmadat és megtaláld azokat a módszereket, amikkel könnyebben és hatékonyabban tudsz megküzdeni vele. Az egyik ilyen mód a MOZGÁS!

De ez a program Rólad szól! Te találod ki és Te állítod össze a saját mozgásprogramodat, hiszen nincs két egyforma ember, így nincs két egyforma megoldás sem. Nincs helytelen válasz! Akármekkora célt kitűzhetsz magad elé és mindenféle mozgás, mindenféle ötlet teljesen rendben van.

A három hónap alatt háromszor lesz szükséged a munkalapra, hogy meg tudd válaszolni a kérdéseket. De arra bíztatlak, hogy lapozgasd minél többször és gondolkodj el a kérdéseken, esetleg válaszold meg Őket amilyen gyakran csak tudod.

Kezdjünk is bele!

M³ TRÉNING

1. Alkalom

1. Kérlek írj pár mondatot a fájdalmadról. Bármit írhatsz, ami eszedbe jut.

2. Mit gondolsz arról, hogy a mozgásnak milyen szerepe lehet a fájdalom kezelésében?

3. Számodra mit jelent a mozgás? Mit szeretsz mozogni? Szoktál rendszeresen mozogni?

4. Előfordult-e az elmúlt pár napban olyan, hogy elkezdtél mozogni és ez felerősítette a fájdalmadat?
Hogyan történt ez? Írd le pár mondatban.
Olyan részletekre is kitérhetsz, hogy hol voltál épp, vagy kivel voltál épp.

5. Volt-e olyan az elmúlt pár napban, hogy érezted a fájdalmad, de a mozgás javított rajta? Ezt is fejtsd ki. Milyen mozgás volt az? És mik voltak a körülmények?

6. Tudnál olyan példákat írni, amikor annak ellenére is mozogtál, aktív voltál, hogy fájdalmad volt közben?

7. Írj legalább 5 olyan tevékenységet, ami most fontos a számodra és amiben a mozgásnak fontos szerepe van. Ezeket írd a „MOZGÁS" oszlopba". A „CÉL" oszlopba írd be, hogy milyen gyakran szeretnéd az adott mozgást végezni. Majd rangsorold őket fontossági sorrendbe. Az 1-es legyen az, ami jelenleg a legfontosabb a számodra, a kettes ami a második legfontosabb és így tovább.

MOZGÁS	CÉL
☐	
☐	
☐	
☐	
☐	
☐	
☐	
☐	
☐	

8. Írd be a táblázatba, hogy jelenleg van-e olyan, ami miatt nehezedre esik mozogni? Majd azt is írd be kérlek, hogy mik azok, amiért mégis megérné mozogni?

MIÉRT NEHÉZ MOZOGNI?	MIÉRT ÉRI MEG MOZOGNI?

9. Az előbb leírt 5 mozgásos cél közül melyik lenne az, amit akár már holnap elkezdesz?

10. Mennyire vagy magabiztos abban, hogy el is kezdesz mozogni már holnap?
Ezt jelöld meg az 1-10-ig skálán. Az 1-es azt jelenti, hogy biztos nem állsz neki, a 10-es pedig azt, hogy 100%, hogy nekiállsz. Miért ezt a számot adtad magadnak?

1 2 3 4 5 6 7 8 9 10

Azért ezt a számot adtam magamnak, mert...

11. Mi segíthet abban, hogy holnaptól többet mozogj?

12. Írd le, hogy hányszor tervezel mozogni a következő héten!

13. Írd le, melyik napokon, és esetleg azt is, melyik napszakban tervezed a mozgást!

14. Írd le, hogy mennyit tervezel mozogni ezeken az alkalmakon!

15. Gondold végig, hogy kik azok, akik szinte mindennap kapcsolatban vannak Veled és tudnának segíteni Neked a terved végrehajtásában. Az ő nevüket írd a „SEGÍTŐIM" oszlopba. Bárkit írhatsz, akit fontosnak gondolsz.

SEGÍTŐIM	HOGYAN TUD SEGÍTENI?
....	....
....	....
....	....
....	....
....	....
....	....
....	....
....	....

16. Írd kérlek a nevek mellé, hogy ki hogyan tudna segíteni Neked. Bármilyen ötletet írhatsz.

17. Írd le, hogy most a beszélgetés után, mit gondolsz, miért lehet hasznos számodra a mozgás?

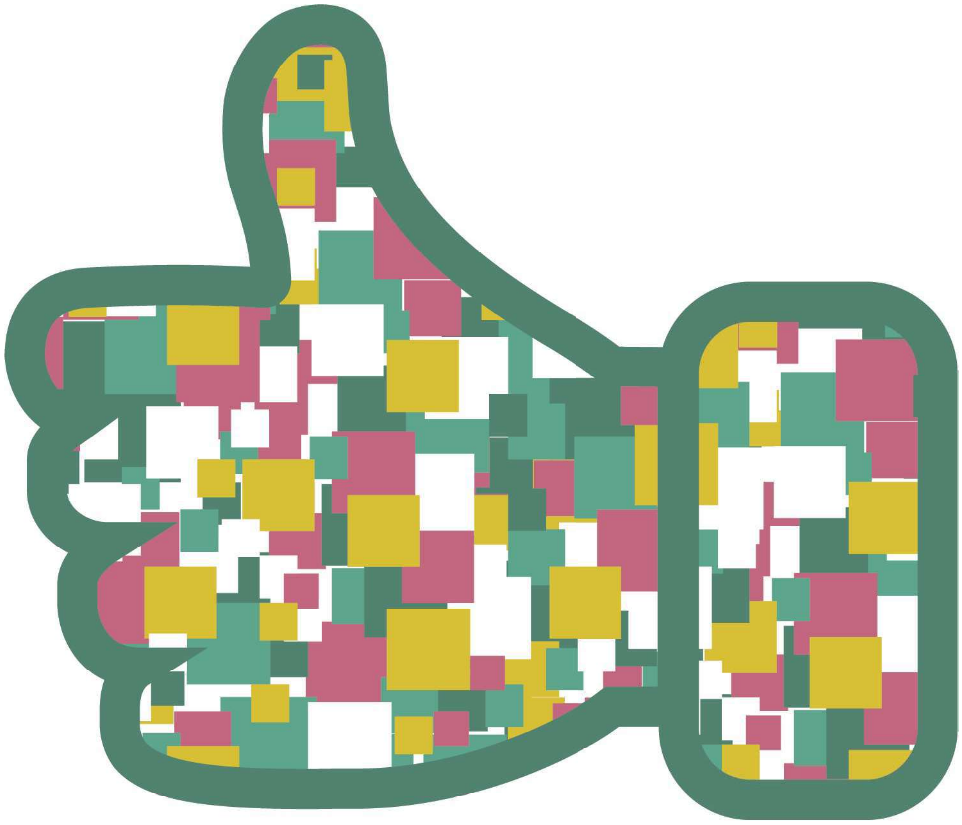

CSAK ÍGY TOVÁBB!
