# Supplementary material for: Novel motivational interviewing‐based intervention improves engagement in physical activity and readiness to change among adolescents with chronic pain
Source: Health Expect. 2024 Mar 31;27(2):e14031. doi: 10.1111/hex.14031 (PMC10982597; doi:10.1111/hex.14031)
Supplement: Supplementary file 2 — Appendix 2. CERT checklist and individual exercise programs. [file HEX-27-e14031-s002.docx]

**Appendix 2.**

The M3 Training program is designed to promote exercise in a person-centered and individualized way using motivational interviewing techniques. Despite the individual customization of the program, some common elements can be found in all the self-made M3 Training exercise programs. The first table shows general themes. The second table lists individual exercise programs and information about previous exercise habits and painful areas. This report has been prepared following the CERT checklist^1^.

**Table 1. Common elements of the M3 Trainings**

| **CERT checklist element** | **Common aspects of the M3 Training** |
| --- | --- |
| Individual/group exercise | Each exercise program was created by teenagers to be performed independently without the direct supervision of the physiotherapist. In some cases, the teens opted to participate in group activities at a gym or exercise facility. Additionally, there were instances where adolescents chose to exercise with their parents. Each of these cases is indicated by the corresponding movement programs. |
| Supervision | There was no direct supervision. Sometimes, the parents were present or supervised the pre-planned exercise activities. |
| Adherence | During the second and third consultations, we asked the teenagers to reflect on their exercise program's progress, both successful and unsuccessful attempts. We also asked parents to reflect on their child's advancement. |
| Motivation | M3 Training (see detailed description in main text **2.4.2. M3 Training**) |
| Progression | During the second and third consultations, either the physiotherapist suggested, or the teen brought up the idea of progression. Progression plans were always based on the teen's motivation and willingness to move forward. If the teenager did not want to make progress, the physiotherapist respected their decision. Maintaining the original amount of exercise was considered a success in such cases. On the other hand, if the adolescent was willing to progress further, the physiotherapist provided information about pacing and gradual loading recommendations. It was recommended that teens keep a 10% increase in their program. This increase could be in the particular exercise's distance, time, or repetition number. |
| Non-exercise elements | Progressive relaxation and breathing exercises. (see **Appendix 3**.) |
| Adverse events | The participants did not report any adverse events related to their exercise program. Due to the nature of chronic pain, we did not count pain experience during the activities as an adverse event. |
| Exercise description | Sometimes, teenagers sought advice regarding home exercise programs. In such cases, the PT suggested using PhysiApp and created a customized movement program that met the teens' specific requests while being appropriate for their pain issues. These instances are noted in Table 2 under the respective individual programs. |

**Table 2. Individual exercise programs**

| ID | Activity history | Pain problem | Individual exercise plan |
| --- | --- | --- | --- |
| A1 | hip-hop dance two times a week (in braces) | wrist pain on both sides; wears a wrist brace most of the time | playing the piano three times a week for 10 minutes (Tuesday, Thursday, Saturday, or Sunday); writing one hour in school every weekday without braces |
| A2 | karate – does not like it; used to be an active teen | mostly lower limb pain and, at times, the entire body. | teen challenges their parent (for fun) to do exercises four times a week for 15 minutes each. (Friday, Saturday, Sunday, Monday), playing basketball in the garden three times for 30 minutes (Friday, Saturday, Sunday) |
| A3 | no sports or regular exercise | headaches | elliptic trainer, 3x (Monday, Tuesday, Thursday), 30-34 minutes each |
| A4 | no sports or regular exercise | headaches | bicycle 1 or 2 times a week, 30 minutes (Monday and Tuesday) |
| A5 | swimming but not regularly | abdominal pain | every morning, 15 minutes of belly exercises to help bowel movements using **PhysiApp**; swimming 1-2 times a week for 1 hour |
| A6 | water polo at a competitive level | headaches | progressive relaxation and breathing exercises three times over the next week |
| A8 | synchronized swimming for four years – stopped doing it | headaches and knee pain | knee strengthening exercises using **PhysiApp** 3 times a week, 30 minutes each (Tuesday, Thursday, and Saturday) |
| A9 | triathlon for seven years – stopped doing it two years ago | headaches | running on Thursday afternoon; minimum one and maximum three times next week for 40 minutes |
| A10 | does not engage in any sports or exercise, as they do not enjoy physical activity | abdominal pain | walking with friends; try brisk walking for 10 minutes each time.  Playing ping-pong once for their mother's sake. |
| A11 | does not engage in any sports or exercise, as they do not enjoy physical activity | headaches | 5 minutes of exercise every day (push-ups, sit-ups, squats, plank, lunges, chest press with 2 kg dumbbells) |
| A12 | no sports or regular exercise | headaches | hiking during the weekends, walking in the evenings for 60 minutes, home exercises for 30 minutes (without equipment, body weight exercises, abdominal)  will try the progressive relaxation and breathing techniques at least once |
| A13 | used to be a competitive swimmer – still swims but not in a competitive way | lower back pain | swimming three times a week but at intervals. To determine the baseline of activity, the teen will stop before the pain increases, rest and, do breathing exercises, and then continue. Doing breathing exercises in stressful situations. As part of their exposure therapy, they will start bending forward daily, even though they previously avoided this motion. Will perform the bending exercises on **PhysiApp**. |
| A14 | used to be a competitive swimmer – but stopped doing it | lower back pain | would like to swim again. Planning to swim two times over the next week, starting with 250 m, which is ten laps. Going to try the breathing exercises. |
| A15 | no sports or regular exercise | neck pain | the teen will accompany her mother to work with the scooter (manual, not electronic). Will try to do home exercises six times a week for 15 minutes each for her neck and the whole body following a videotape. Did not want the help of the application. |
| A16 | no sports or regular exercise | abdominal pain | biking in the forest Friday afternoon for 20-30 minutes |
| A17 | no sports or regular exercise | abdominal pain | general strengthening exercises in **PhysiApp** minimum of three and maximum of 4 times a week for 10 minutes. Most likely in the mornings, less likely in the evenings. Might try running later. |
| A18 | used to swim – stopped doing it; does not like sports | headaches | home exercise program four times a week for 15 minutes. (Exercises: squats, lunges, push-ups, stretching the hamstrings, sit-ups) |
| A19 | no sports or regular exercise | lower limb pain | elliptic trainer three times 5 minutes, plus ten push-ups three times a week |
| A20 | used to dance but stopped; unable and unwilling to attend school due to pain | headaches | will try to go to school at least one day; dancing every evening for 15 minutes. Would like to run again - interval training is offered - will try it in school. |
| A21 | athletics – running; does not do it now due to pain | abdominal pain | starting running again - interval training is offered and created. will try it three times next week |
| A22 | does not engage in any sports or exercise, as they do not enjoy physical activity | abdominal pain | as climbing interests them, they will try it once next week- never did it, want to try it |
| A23 | plays football weekly, but not at a competitive level | headaches | breathing and progressive relaxation two evenings in bed before sleep |
| A24 | no sports or regular exercise | headaches | walking 5 km on their own or with parents on Tuesday, Wednesday, and Sunday afternoons |
| A25 | teen has always been active but cannot do any sport now due to pain | lower limb pain | starting to do the following exercises four times next week:  standing push-up at the wall for five reps; stationary bike 6 minutes; walking up and down stairs at home two times more than before; squats with hand support, a minimum of six reps and a maximum of eight reps; ankle flexion and extension of ten times in sitting or lying; heel raises ten times; hula-hoop for 5 minutes |
| A26 | used to be a professional dancer – stopped and has not been doing sport since | headaches | exercises in the gym three times a week for one hour. following their previous routine, using gym equipment and free weights (dumbbells). Does not need the application and would like to create its own program. The PT provided details regarding the concepts of progressive loading and pacing. |
| A27 | folk dance – 2 times a week | headaches | boxing on Kinect with parent 3-4 times a week (Tuesday, Thursday, Friday, and Saturday) - walking two times for 30 minutes with parents |
| A28 | no sports or regular exercise | lower back pain | exercises on **PhysiApp** for 10-15 minutes a day |
| A28 | no sports or regular exercise | abdominal pain | running one time for 20 minutes |
| A30 | climbing 2-3 times a week – actively participating | abdominal pain | breathing exercises in school if experiences belly pain, 10-15 push-ups every day to enhance climbing performance, one-time biking with parents |
| A31 | used to do ballet – stopped, do not do any exercise or sport now; unable and unwilling to attend school due to pain | headaches | stretching 30 minutes every second day in the morning – focusing mostly on hip adductors as the teen would like to be able to do seated split again. Will try the ballet routine for adductor stretch instead of using an application. |
| A32 | no sports or regular exercise | hand and wrist pain, difficulties in writing in school | home exercises 3-5 times a week, 30-60 minutes following the exercise program in **PhysiApp** for hand strength and general strength. Adolescent wants to try out some balance exercises for fun. |
| A33 | Professional dancer for seven years – cannot participate as regularly as before | mainly knee pain, but sometimes the whole body and joints | strengthening exercises for the knee and whole body (explicit focus on abdominal and upper arm muscles as per the wishes of the teenager) following the exercises in **PhysiApp**, minimum one and maximum 2 times a week Friday, Sunday, or Monday |

1. Slade SC, Dionne CE, Underwood M, Buchbinder R. Consensus on Exercise Reporting Template (CERT): Explanation and Elaboration Statement. *Br J Sports Med*. 2016;50(23):1428-1437. doi:10.1136/bjsports-2016-096651
