# Supplementary material for: Novel motivational interviewing‐based intervention improves engagement in physical activity and readiness to change among adolescents with chronic pain
Source: Health Expect. 2024 Mar 31;27(2):e14031. doi: 10.1111/hex.14031 (PMC10982597; doi:10.1111/hex.14031)
Supplement: Supplementary file 3 — Appendix 2.1 Individual exercise program (A5). [file HEX-27-e14031-s008.pdf]

1 Set / 10 Reps / 2 min duration

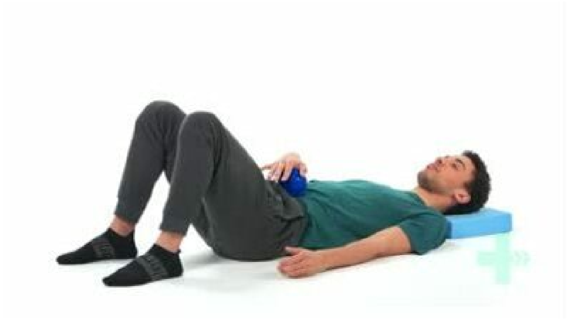

1. Abdominal massage

Lie on your back with your knees bent and your feet flat on the floor.
Using a massage ball, press firmly in a clockwise direction, massaging up the outer edges of the right side of your abdomen, and down the outer edge of the left side.
Pause and hold over any tender spots.

1 Set / 10 Reps

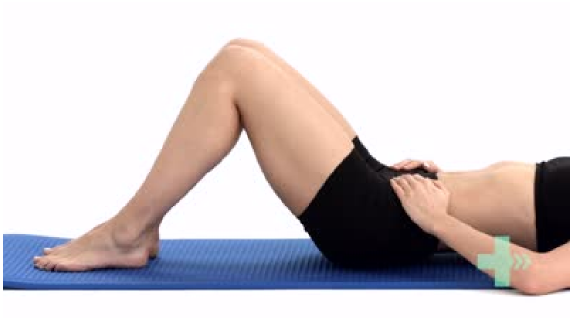

2. Neutral spine setting, with core/pelvic floor activation, supine; 01

Lie on the floor with your legs bent.
Place your hands on your hip bones at the front and tuck your chin in.
Tilt your hips to roll the tail bone under and flatten the lower back, then lower your tail bone and arch your lower back.
Neutral spine is around mid way between these two positions.
Hold this position by activating your core stability muscles in your tummy and pelvic floor.
Exercise Tip – imagine your pelvis is a bowl of water and you are tipping water out of the front and the back.

1 Set / 10 Reps / 2 s hold

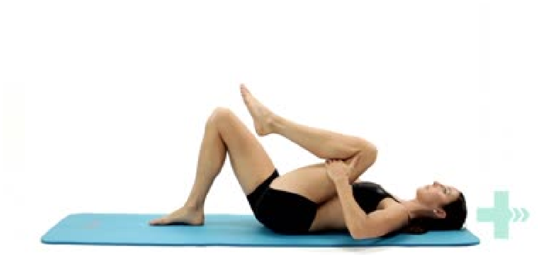

3. Hip flexion AAROM end range, supine

Bend your knees so that the feet are flat on the floor.
Reach underneath your knee and pull the knee in towards your chest as far as you can go comfortably.
Ensure you grab behind the thigh and not on top of the knee to avoid compressing the knee joint.
Hold this position.

1 Set / 10 Reps / 2 s hold

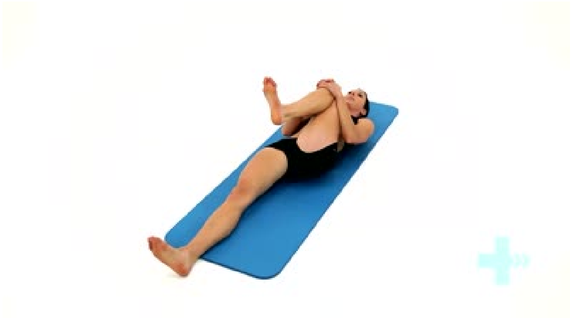

4. "Knee hugs" Hip/lumbar extensors stretch, single leg, supine; 02

Lie on your back with your legs straight.
Hug the knee of the affected leg in to your chest as far as you can go comfortably.
Pull the knee in towards the mid line of your body to increase this stretch.

1 Set / 10 Reps / 2 s hold

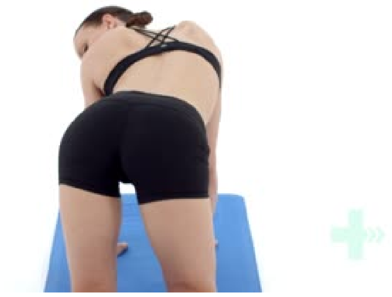

5. Trunk side bending AROM, looking toward buttock, quadruped; 02

Start on your hands and knees, with your hands under your shoulders, and knees under your hips.
Look over your shoulder towards your right buttock whilst bending from your middle to push your hips around to the right.
Return to the start position.
Look over your left shoulder towards your left buttock whilst bending from your middle to push your hips around to the left.
Return to the start position.

1 Set / 10 Reps / 2 s hold

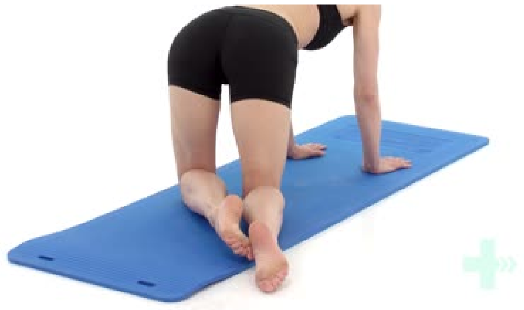

6. Trunk side bending AROM, quadruped

Start on your hands and knees, with your hands under your shoulders, and knees under your hips.
Lift your feet up and turn them both to one side.
Move your gaze and upper body to look round to your feet, creating a “C” shape with your spine.

1 Set / 10 Reps / 2 min duration

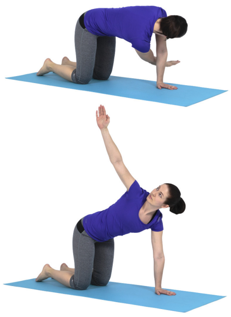

7. Thoracic Spine Rotation

On all fours, hands under your shoulders and knees under your hips. Spine in neutral position.

Reach one arm under the other armpit reaching beyond the opposite knee and then bring the arm back and reach towards the ceiling. Follow the arm movement with your head and gaze.

Note: Focus on the movement from your thoracic spine.

1 Set / 10 Reps

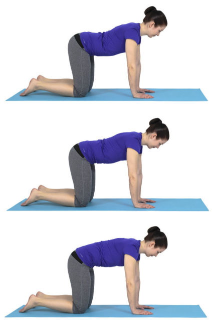

8. Lower Back Flexion/Extension

On your hands and knees, hands under your shoulders, and knees under your hips.

Maintaining neutral position in your upper and mid-back, round and arch your lower back by tilting your pelvis.

1 Set / 10 Reps

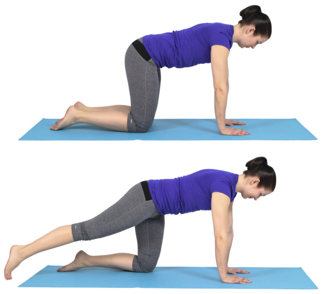

9. Hip extension in four point kneeling (toes on the floor) - movement control

On your hands and knees, hands under your shoulders and knees under your hips.
Spine is in neutral position.
Straighten and lift one leg.
Lift only as high as you can control the position of your spine.
Repeat with your other leg.
Note:
- Keep your hips level and lower back in neutral position.
- Try to keep the weight shift sideways as small as possible.

1 Set / 10 Reps

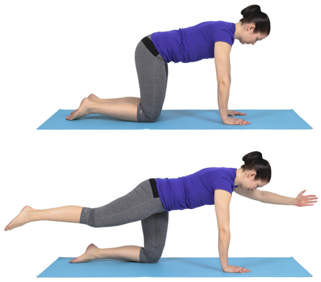

10. Arm and Leg Lift (Bird-dog)

On your hands and knees, hands under your shoulders and knees under your hips. Spine is in neutral position.

Lift one arm and opposite leg. Lift only as high as you can control the position of your spine.

Note:
- Keep your lower back in neutral position.
- Try to keep the weight shift sideways as small as possible.
