# Supplementary material for: Novel motivational interviewing‐based intervention improves engagement in physical activity and readiness to change among adolescents with chronic pain
Source: Health Expect. 2024 Mar 31;27(2):e14031. doi: 10.1111/hex.14031 (PMC10982597; doi:10.1111/hex.14031)
Supplement: Supplementary file 4 — Appendix 2.2 Individual exercise program (A8). [file HEX-27-e14031-s010.pdf]

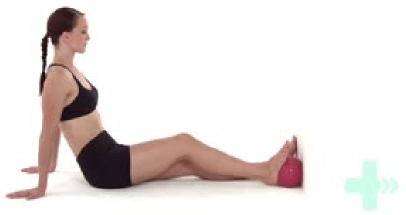

2 Sets / 12 Reps / 1 s hold

**1. Knee terminal extension strengthening, pushing heel into ball, sitting**

Sit with your legs straight out in front of you.
Place a small ball against a wall.
Put the foot of your affected leg flat up against the ball, with your knee slightly bent.
Keep your knee pointing directly upwards.
Push the heel of your foot in to the ball, trying to straighten your knee.
Use the resistance of the ball to push against so you feel the movement.

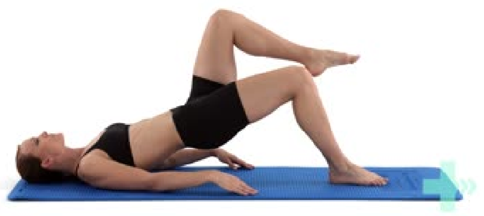

2 Sets / 12 Reps / 1 s hold

**2. "Bridge, single leg" Core/gluteals strengthening; 01**

Lie on your back.
Bend one leg upwards, placing the foot on the floor.
Draw your other leg up to the same position, maintaining a hips width between your legs.
Lift one foot slightly off the floor.
Using the effort of your remaining foot, lift your hips up into a bridge until you have a straight line from your shoulders to your knees.
Lower back down and repeat.
Keep your pelvis level throughout this exercise.

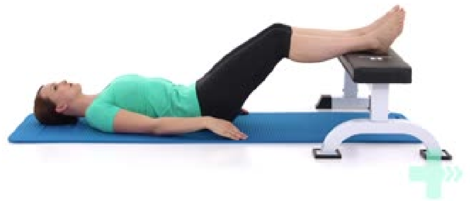

2 Sets / 12 Reps / 1 s hold

**3. "Bridge" Core/gluteals strengthening, feet on bench**

Lie on your back with your heels rested securely on a bench.
Ensure your knees and feet are hips distance apart with your knees pointing to the ceiling.
You should have your knees bent to approximately 45 degrees.
Tighten your abdominal and buttock muscles and roll your tail bone up from the floor.
Continue this movement, lifting your hips directly up to the ceiling until you have a straight line from your shoulders to your knees.
Keep your neck and shoulders relaxed.
Control the movement as you lower your hips back down to the floor.
Your abdominal muscles should remain engaged until your lower back reaches the floor.

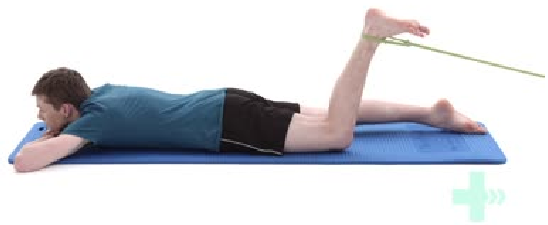

2 Sets / 12 Reps

**4. "Hamstring curl" Knee flexion strengthening, with band, prone**

Lie on your front with a resistance band tied around one ankle and secured behind you.
Bend the knee against the resistance from the band, moving your heel towards your buttocks.
Control the pull of the band as you lower the leg back down.

*Kösd ki a gumiszalagot és a másik végét rögzítsd a bokád körül. Feküdj hasra és lassan hajlítsd be a térded a szalag ellenében, majd lassan nyújtsd ki.*

1 Set / 2 Reps / 35 s hold

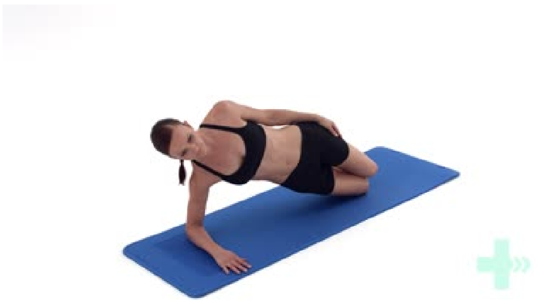

**5. "Plank, side (low)" Core/scapular strengthening isometric, on knees**

Lie on your side and prop yourself up on your elbow.
Bend your knees and lift your hips off the mat until you have a straight line from your knees to the top of your head.
Hold this position for as long as you can.

2 Sets / 12 Reps

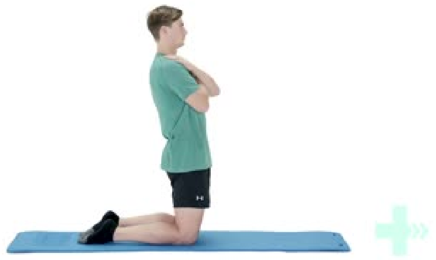

**6. "Nordic curl, reverse" Iliopsoas/quadriceps strengthening eccentric**

Kneel up high on a mat.
Your legs should be hips distance apart.
Cross your arms over your chest and activate your buttock and abdominal muscles.
Keeping your hips pushed forward in line with your body and thighs, slowly lean your whole body back over your calves.
Do not allow your hips to drop backwards from your body and thighs.
Control the movement as you return to the start position and repeat at a slow to steady pace.

2 Sets / 12 Reps

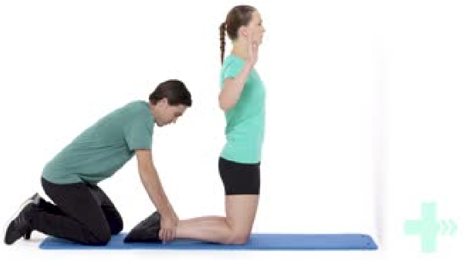

**7. "Nordic curl" Hamstring strengthening eccentric, into push up, against wall - with partner**

You will need an assistant for this exercise.
Begin by kneeling upright on the floor facing a wall.
Ask your assistant to hold onto your heels for stability.
Keep your hips in line with your knees, maintain a neutral spine and engage your deep abdominal muscles throughout this exercise.
Slowly lean your upper body towards the wall by opening up the angle at your knees.
At the end of the movement, place your hands on the wall to push yourself back up to the start position.
Repeat.
You can increase the difficulty of this exercise by starting further away from the wall.

1 Set / 2 Reps / 60 s hold

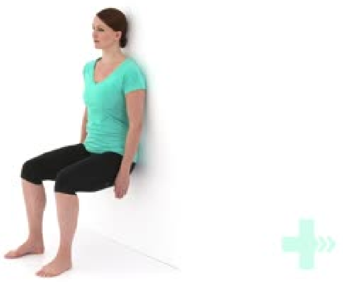

**8. "Squat, on wall" Lower body strengthening isometric, 90/90**

Stand up straight with a wall positioned behind you.
Lean your back and buttocks against the wall, then walk your feet forward.
Slide down the wall until you reach a 90 degree angle at your hips and knees.
Ensure your back and buttocks remain in contact with the wall.
Hold this position.

1 Set / 2 Reps / 60 s hold

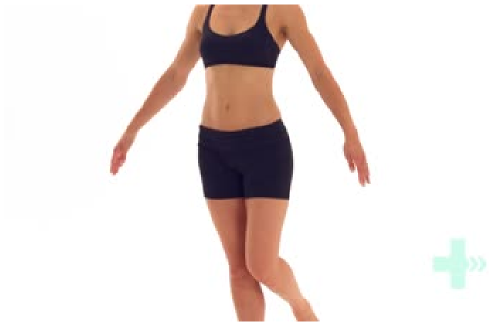

**9. Balance, single-leg, eyes open, near support; 02**

Balance on your symptomatic leg for as long as you can.
You may want to be close to a solid object to hold on to if needed.
Do not rest your bent leg on the stance leg.

2 Sets / 12 Reps

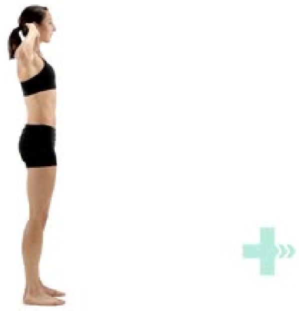

**10. "Hip hinge, good mornings" Posterior chain strengthening, hands behind head**

Stand with your feet shoulder-width apart and place your arms behind your head.
Tilt the pelvis so that you push your tail bone out, and tighten the abdominal muscles.
Lean forward as you keep your weight in the heels, pushing your hips directly back behind you. It is vital you keep the weight in your heels keep your spine completely straight.

2 Sets / 12 Reps

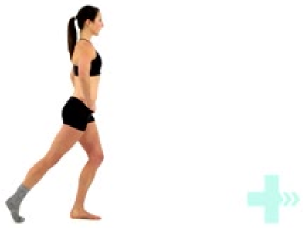

**11. "Squat, single leg" Lower body strengthening, with hip extension slide**

Wear a sock on your good leg and stand up tall.
Slide this leg back behind you, bending the affected leg, making sure your knee travels directly over your toes.
Straighten back up, sliding your foot back in before your repeat.
Keep your chest up straight and heel on the ground throughout this exercise.

2 Sets / 12 Reps

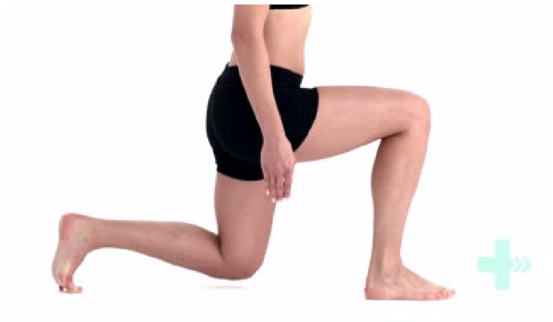

**12. Lunge, on the spot**

Take a large step forwards on your affected leg.
Drop your hips directly down between your two feet.
Allow both legs and hips to bend, so that your knees are at 90 degrees, your back heel comes off the floor and the shin of your front leg is vertical.
Push back up to the starting position and repeat.
Make sure your knees travel directly forwards over your toes at all times.

2 Sets / 20 Reps

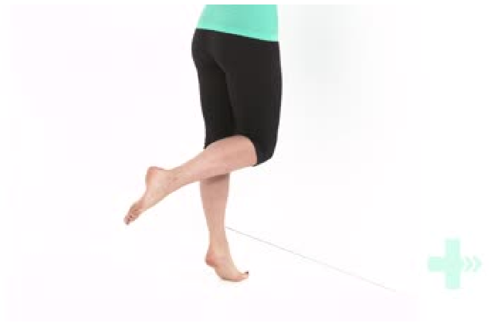

**13. "Heel raises" Ankle plantar flexion dynamic strengthening, single leg, near wall**

Stand up straight facing a wall.
Place both hands on the wall in front of you for support.
Stand on one leg.
Rise up onto the ball of your foot then lower your heel back to the floor.
Continue this movement at a fast pace.

2 Sets / 12 Reps / 1 s hold / 2kg weight

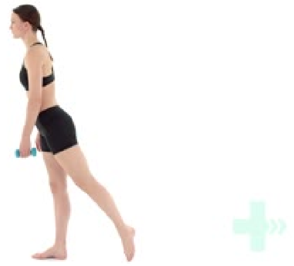

**14. "Hip hinge, single leg" Posterior chain strengthening, with dumbbell; 01**

Stand up straight balancing on your affected leg.
Hold a weight in your opposite hand.
Lift the other leg and hold it straight behind you.
Bend forwards at the hip so that your body moves towards the foot on the floor.
Your elevated leg should move backwards at the same rate.
Make sure you move through the hip and that you keep your spine in neutral.
You may feel the back of your legs working particularly on the way down.
Move at a steady rate before you slowly return to the standing position again.

2 Sets / 12 Reps / 1 s hold

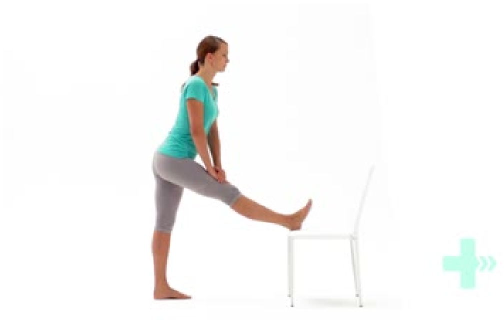

**15. Hamstring stretch, standing foot on chair**

Place the foot of your affected leg onto a chair or step.
Keep your knee straight and foot pointing ahead.
Keeping your back straight, tip forwards from your hips, pushing your buttocks out behind you until you feel a stretch down the back of your thigh.
Hold this position.

2 Sets / 12 Reps / 1 s hold

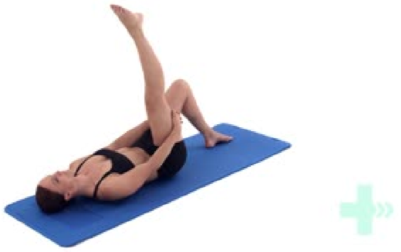

**16. Hamstring stretch, straightening leg, supine feet standing; 02**

Lie on your back and bend your affected leg in towards you.
Interlace your fingers behind the thigh to firmly hold the leg as you straighten out your knee, feeling the stretch behind your thigh.
