# Supplementary material for: Novel motivational interviewing‐based intervention improves engagement in physical activity and readiness to change among adolescents with chronic pain
Source: Health Expect. 2024 Mar 31;27(2):e14031. doi: 10.1111/hex.14031 (PMC10982597; doi:10.1111/hex.14031)
Supplement: Supplementary file 5 — Appendix 2.3 Individual exercise program (A13). [file HEX-27-e14031-s007.pdf]

1 Set / 5 Reps / 4 s hold

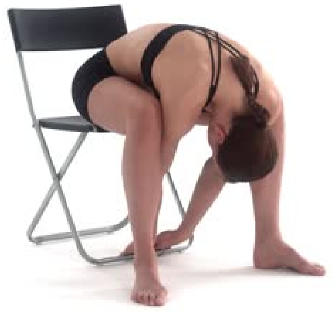

1. "Spinal roll down" Cervical/trunk extensors stretch, sitting legs apart

Sit on a chair and slowly bend forward to glide your arms in between your knees under the chair.
Control the pace of the forward bend by gently pressing your arms against your inner thighs.
Reach under the chair with both arms and allow your lower back to stretch.
Breathe in and out during the stretch.
To come back up, bring one hand on your foot and use this as a support to bring your other hand just above your knee.
Use the hand on your knee to push yourself back into a seated position.

Ülj le egy székre és lassan csúsztasd a karjaidat lefelé a talaj felé a térdeid között egészen a szék alá. Te kontrollálod a sebességet. Folyamatosan lélegezz a hasadba. Orron szívd be lassan, majd lassan fújd ki. Lazítsd el a hátadat, amennyire csak tudod. Maradj lent 2 hosszú légvételig, majd ha szükséges, tedd a tenyereidet a térdeidre és a kezeid segítségével egyenesedj ki. Ismételd a feladatot 5x, naponta többször.

4 Sets / 5 Reps

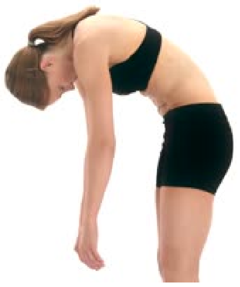

2. "Spinal roll down" Cervical/trunk extensors stretch, standing - to mid leg

Stand with your feet hips width apart.
Breathe in and roll your head and upper chest downwards from the top of your spine by bringing your chin towards your chest opening your neck.
Curl into yourself, gathering your tummy allow your arms and shoulder blades hang freely on either side.
When your hands are around knee level, take a breath in.
As your breath out, gather your tummy in.
Roll your body back up, pulling from the lower back muscles.
Let your shoulders hang loosely to the side through the entire movement.
At the end of the movement unfold the neck to and bring your shoulder blades back to return to the start position.

Állásból indulva lassan döntsd előre a fejed, majd gömbölyítsd ki a nyakad, a hátad és végül a derekadat. Hajolj előre addig, amíg nem túl kellemetlen. Próbáld elengedni magad, lógni kicsit. Folyamatosan lélegezz a hasadba. Orron szívd be lassan, majd lassan fújd ki. Lazítsd el a hátadat, amennyire csak tudod. Maradj lent 2 hosszú légvételig, majd ha szükséges, tedd a tenyereidet a térdeidre és a kezeid segítségével egyenesedj ki. Ismételd a feladatot 5x, naponta többször.

1 Set / 15 Reps / 2 s hold

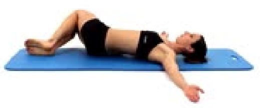

3. Lower trunk rotation AROM, to comfort, supine feet standing; 01

Lie on your back with your knees bent and your feet flat on the floor.
Extend your arms out to the sides and keep your shoulders on the mat at all times.
Keeping your knees together, drop them down to one side, rotating your torso.
Return to the starting position and allow your knees to fall to the opposite side.
Only drop your knees as far as you go comfortably.
You may want to hold the stretch on each side.

Feküdj a hátadra. A lábaid legyenek talpon. Lazán döntsd ki a két térdedet jobbra, majd balra. Nem kell erőltetni, a cél, hogy ellazulj.

1 Set / 15 Reps / 2 s hold

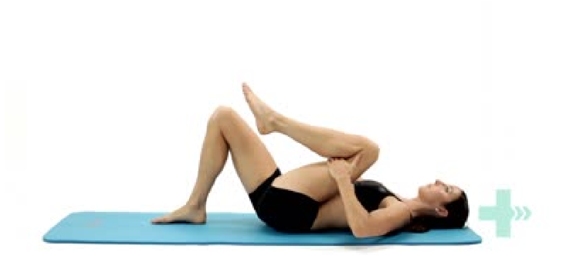

4. Hip flexion AAROM end range, supine

Bend your knees so that the feet are flat on the floor.
Reach underneath your knee and pull the knee in towards your chest as far as you can go comfortably.
Ensure you grab behind the thigh and not on top of the knee to avoid compressing the knee joint.
Hold this position.

Háton fekvésben húzd egyik, majd másik térdedet a hasadhoz. Tartsd a mellkasodhoz közel a térded 2 másodpercig, majd tedd le. Felváltva végezd a feladatot.

1 Set / 20 Reps / 1 s hold

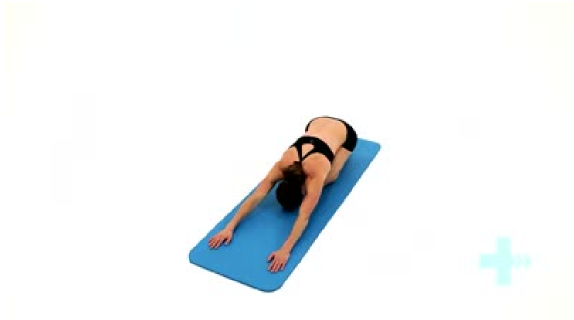

5. "Child's pose" Shoulder/trunk extensors stretch, quadruped; 01

Get onto your hands and knees, and drop your buttocks back onto your heels.
Stretch your hands forwards, dropping your head between your shoulders towards the floor.
You will feel this stretch through your back and upper arms.

Négykézláb helyzetből ülj hátra a sarkaidra úgy, hogy a karjaiddal előre megnyújtózol a talajon. Próbálj egyre közelebb ülni a sarkaidhoz. Nem kell erővel letolni magad, inkább "ringatózz" előre-hátra.
