# Supplementary material for: Novel motivational interviewing‐based intervention improves engagement in physical activity and readiness to change among adolescents with chronic pain
Source: Health Expect. 2024 Mar 31;27(2):e14031. doi: 10.1111/hex.14031 (PMC10982597; doi:10.1111/hex.14031)
Supplement: Supplementary file 6 — Appendix 2.4 Individual exercise program (A17). [file HEX-27-e14031-s004.pdf]

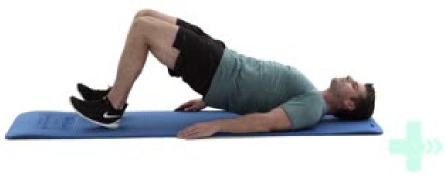

2 Sets / 8 Reps / 2 s hold

1. "Bridge" Core/gluteals strengthening isometric, toes up

Lie on your back and bend your knees at 90 degrees.
Pull your toes in towards you and rest your weight on your heels.
Now tuck in your navel and lift your pelvis off the floor.
Tighten your buttocks and your abdominal muscles and keep breathing normally.
Make sure your back is in line with your thighs, so you can draw an imaginary line from your shoulders through your back, pelvis and knees.
Make sure you keep your pelvis level during this exercise, hold for the required time and then lower back down in a controlled manner.

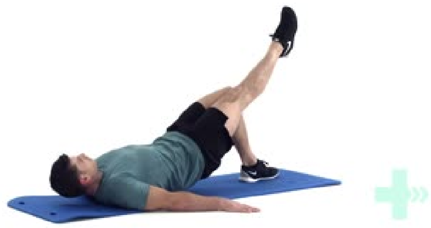

2 Sets / 8 Reps / 2 s hold

2. "Bridge" Core/hip stabilization, straightening leg (alternate), head lifted

Start position is lying on the back with the hips raised off the floor and body supported by the upper back and feet, keep the head off the floor with the chin tucked in.
Lift one leg and straighten at the knee.
Keep the leg aligned with the trunk.
Do not let the pelvis sag down to the side.
Hold this position, then switch legs without lowering the hips, as long as your form remains perfect, continue on switching over between legs.
Advanced techniques include placing the arms overhead and the heels further from the body when commencing the exercise.

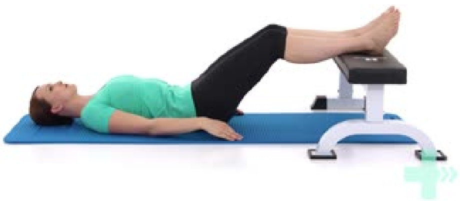

2 Sets / 8 Reps / 2 s hold

3. "Bridge" Core/gluteals strengthening, feet on bench

Lie on your back with your heels rested securely on a bench.
Ensure your knees and feet are hips distance apart with your knees pointing to the ceiling.
You should have your knees bent to approximately 45 degrees.
Tighten your abdominal and buttock muscles and roll your tail bone up from the floor.
Continue this movement, lifting your hips directly up to the ceiling until you have a straight line from your shoulders to your knees.
Keep your neck and shoulders relaxed.
Control the movement as you lower your hips back down to the floor.
Your abdominal muscles should remain engaged until your lower back reaches the floor.

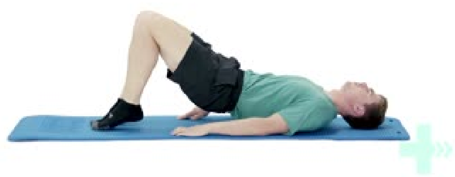

2 Sets / 8 Reps / 2 s hold

4. "Bridge" Core/hip stabilization, 1-2-3 kicks (alternating)

Lie on your back with your legs bent and feet flat on the floor.
Your knees and feet should be hips distance apart.
Tighten your buttock and abdominal muscles and lift your hips up into a bridge.
Keeping your hips level and high, raise your heels off the floor a little.
Straighten one leg out in front of you so that it is in line with the rest of your body.
Next, rapidly kick the other leg out in front of you whilst simultaneously bringing your first foot back to the floor.
Continue alternating legs for a count of 3, and then hold.
1-2-3-hold.
1-2-3-hold.
Continue.

2 Sets / 8 Reps / 2 s hold

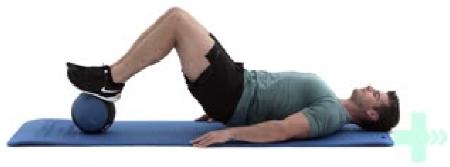

5. "Bridge" Core/hip stabilization, straightening leg (alternate), feet on ball

Lie on your back with your knees bent and a football under your feet.
Tuck in your navel and your pelvic floor and draw your pelvis inwards and up.
Hold this position and try to extend one knee at a time.
Make sure you keep your pelvis level.
Hold this position for the required time and then return to the start position in a slow and controlled movement.

2 Sets / 8 Reps

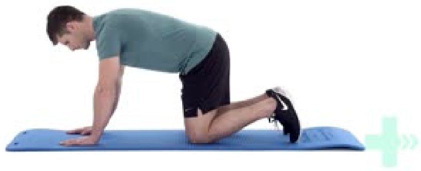

6. "Bird dog" Core/abdominal stabilization; 03

Kneel down on the floor on your hands and knees in the all-fours position.
Keep your back straight throughout this exercise and perform at a slow controlled pace.
Contract the abdominal muscles by pulling your naval in towards your spine and stretch one leg out to the rear, at the same time extend the arm on the opposite side of your body out towards the front.
Do not rotate the trunk and keep your back straight as you extend your arm and leg.
Hold and then return to the starting position.
Then switch to the other arm and leg.
Do two to three repetitions on each side.
Perform this exercise at a slow controlled pace.

2 Sets / 10 Reps

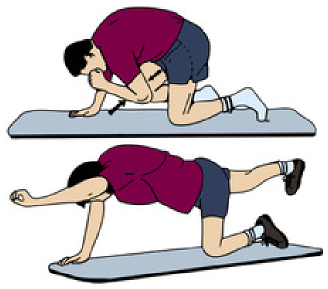

7. Elbow to knee bird dog, Core/abdominal stabilization

Start on your hands and knees, with your hands under your shoulders, and knees under your hips.
Tighten the abdominal core muscles.
Flex the opposite leg and opposite arm simultaneously, to have your knee and elbow meet.
Then extend the leg and the arm simultaneously, making sure you maintain good control in your torso.
Do not allow your body or hips to rotate.
Repeat on the same side for the designated number of repetitions.

2 Sets / 8 Reps / 2 s hold

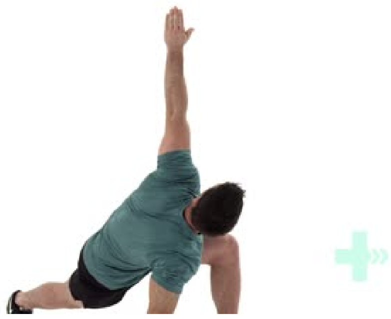

8. Iliopsoas stretch, with trunk rotation, deep split stance; 03

Stand tall with your arms at your side.
Step backward into a lunge with your right foot.
Place your right hand on the ground and your left elbow to the inside of your left foot.
Hold this stretch for a moment and rotate your left arm and chest to the ceiling..
Hold again for a couple of seconds and bring your left arm down and reach it across under your torso to the opposite side.
Return to standing repeat the movement on the opposite side and repeat for the prescribed number of repetitions.

2 Sets / 8 Reps

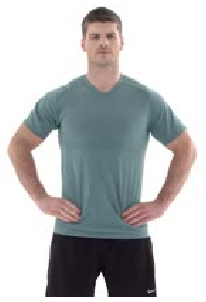

9. Lunge, forward (alternate); 01

Stand upright with your legs at shoulder-width apart and your hands on your hips with the fingers facing in towards your stomach.
Take a medium sized pace forward and then enter into a lunge by lowering your body downwards using your legs.
Allow the forward knee to bend until your thigh is parallel to the floor making sure you keep your back straight.
Return to the starting position by springing up off the front leg.
Repeat for the other leg.
Keep your abdominals tight and your feet shoulder-width apart throughout the exercise.
Perform this exercise at a slow controlled pace.

2 Sets / 8 Reps

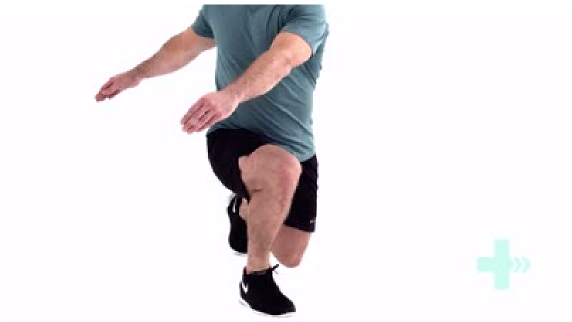

10. Lunge, forward, with trunk rotation, toward front leg (alternate)

Start Position is standing upright with the legs one pace apart.
Perform a full forward lunge by taking a large pace forward so the thigh of the front leg is perpendicular to the trunk and the rear leg is stretched out with the toes on the floor, make sure to keep the trunk upright and the abdominals tight.
Rotate the trunk to the same side as the forward leg and sink further into the lunge position. A stretch should be felt in the hip flexors of the rear leg. Pause in this position for 1- 2 seconds only.
Rise out of the lunge using the power of the front leg, then step through to perform the exercise on the opposite leg.

4 Sets / 10 Reps

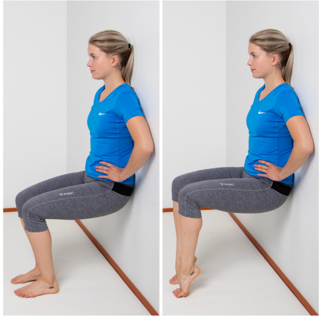

11. Heel Raise in Wall Sit

Start by standing with your back flat against a wall and your feet hip-width apart. Move your feet away from the wall and squat down so that your hips and knees are bent and lower legs are perpendicular in relation to floor.

Press the balls of your feet on the floor and rise onto your toes. Lower the heels back on the floor and repeat.

10 sarokemelésig tartsd - 4x ismételd

2 Sets / 8 Reps

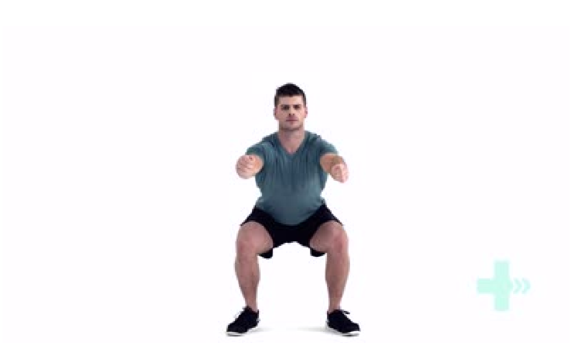

12. Jumping, on the spot, into a squat, with arm swing

Start position is standing with the feet shoulder width apart.
Lower into a squat with the knees aligned over the toes. The heels should be on the floor with the back straight and the head upright eyes looking forward.
Leap upwards out of the squat by swinging your arms behind you and extending through the hips, the knees and the ankles to jump as high as possible. Use the motion of the arms swinging forward to carry to the leap upwards.
Land back into the squat position with the knees bent to absorb the impact through the leg and hips.

2 Sets / 8 Reps

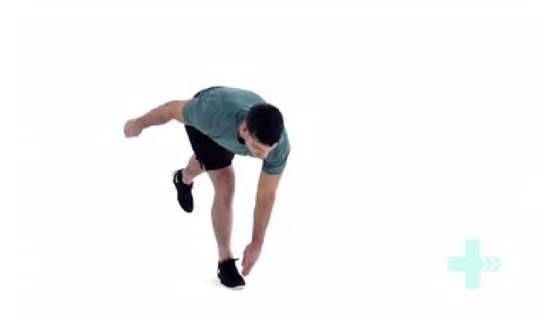

13. Balance, single-leg, reaching to floor, hip hinge position; 02

Start position is standing with one leg just behind the other at shoulder width apart.
The forward leg is the stance leg and the rear leg is one large pace behind the body with the heel raised and toes lightly touching the floor.
Stretch the rear leg backwards whilst counter-balancing with a forward lean of the trunk, and at the same time reach forward with the arm on the same side as the rearward leg and try to touch the floor.
The stance knee should remain centred over the ball of the foot, do not let the knee waiver from side-to-side.
Switch legs and perform the same movements for the other side, if the right leg is stretched back then the right arm is reaching forward.

2 Sets / 8 Reps

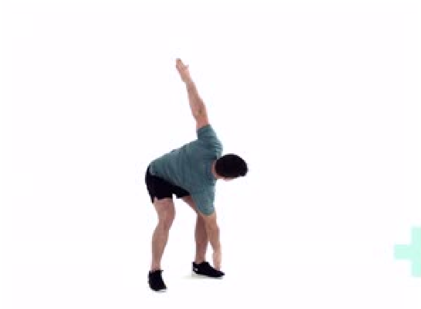

14. "Windmill" Trunk rotation strengthening; 02

Start position is standing in a wide stance with the arms stretched out to the side at shoulder level height.
Move into a semi-squat and hold when arms can touch the floor, then rotate at the hips and the trunk to reach the arms toward the opposite foot.
Stay balanced, with slightly more weight on the side of the reach and keep the head and chest up and aligned.

2 Sets / 8 Reps

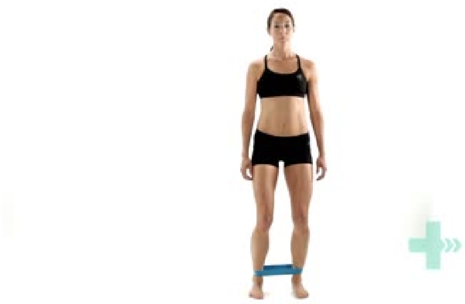

15. Hip abduction strengthening, with band (low), walking to side

Place a band around your ankles and gather some tension.
Side-step keeping constant tension on the band.
Make sure you do not bring your feet too close together and keep your toes and knees pointing forwards.

10 lépés oda-vissza 2x

2 Sets / 15 Reps

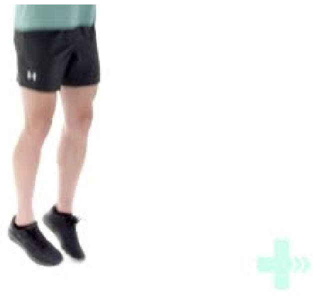

16. Jumping jacks, quickly

Stand up straight with your feet together and with your arms by your sides.
When ready, jump upwards as you open your legs apart and raise your hands above your head in an outward direction.
Land lightly on the balls of your feet before immediately springing back into the air to return to your start position.
Repeat rapidly.

2 Sets / 8 Reps

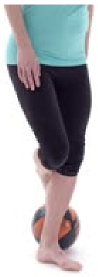

17. Balance, single-leg, rolling ball 360°, around stance leg

Stand up straight and place a ball under one foot.
Roll the ball completely around your stance leg, so that you end up in the starting position.
This exercise can help to strengthen the leg you are standing on, and help with your balance and coordination.

2 Sets / 12 Reps

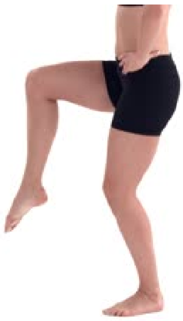

18. Hopping, on the spot, hold (alternate)

Stand on one leg with your other hip elevated at 90 degrees.
Spring up off the floor, landing softly on your other leg, and the first leg elevated to 90 degrees.
Control your balance before you hop back to the initial leg and repeat.

2 Sets / 20 Reps

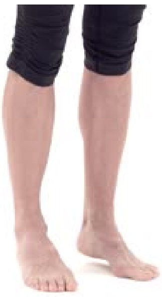

19. "Heel raises" Gastrocnemius strengthening, standing

Start in a balanced stance with your feet shoulder width apart and then raise yourself up on your toes as high as possible.
Return back to the starting position.

2 Sets / 8 Reps / 1 min duration

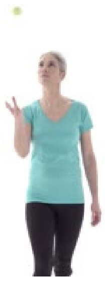

20. Balance, tandem walking, tossing ball

Stand up straight with a clear path in front of you.
Imagine a straight line on the floor extending away from you, or place a straight line of tape along the floor.
Walk along this line on the floor as if you are walking a tightrope.
Throw a tennis ball up into the air and catch it again whilst continuing to walk in a straight line.

2 Sets / 8 Reps / 2 s hold

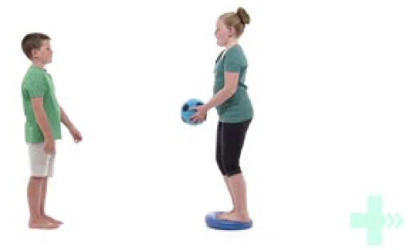

21. Balance, standing, throwing/catching ball, on wobble cushion

Stand up straight with the wobble cushion in front of you.
Hold onto a strong support surface if required.
Step onto the cushion.
Gain your balance.
Once you have got your balance, try throwing a ball into the air and catching it again.
You could also throw and catch with a partner.

2 Sets / 8 Reps

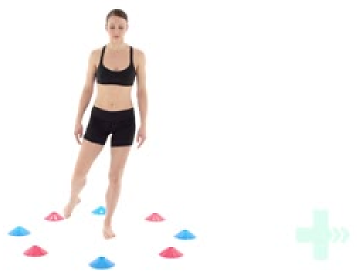

22. Balance, single-leg, touching cones x 8, with opposite foot

Stand up straight on your affected leg.
Place targets around you on the floor at 8 different points like a star.
Balance on your affected leg while trying to slowly touch your elevated foot to each target.
Ensure you keep good control in your stance leg with your knee travelling directly forwards over your toes, rather than inwards.

2 Sets / 8 Reps / 1 min duration

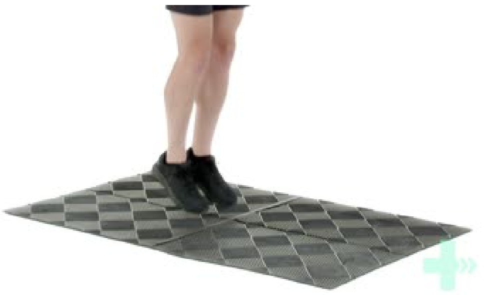

23. Jumping rope

Stand up straight with one end of a jump rope in each hand.
Engage your deep abdominal muscles.
With your hands close together in front of you, begin to build momentum in the rope by swinging it from side to side in a figure of 8 motion.
When ready, separate your hands, tuck your bent elbows into your sides, and turn your forearms away from one another to create a large arc to jump through.
Maintain the momentum in the rope through a flicking action at your wrists, with minimal movement from your arms.
Stay light and springy on the balls of your feet as you begin small two footed jumps over each passing of the rope.
Maintain a fluid circular motion coordinating your wrists and feet, and continue at a steady rhythmic pace.

4 Sets / 8 Reps

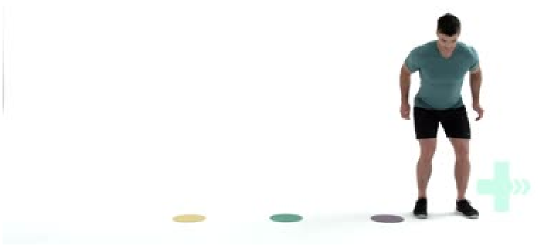

24. Jumping sideways

Stand tall with your arms bent 90 degrees and your forearms facing upwards.
Drive your elbows back and drop into a squat by pushing your hips back and bending your knees.
Immediately push off the floor jumping to the side as far as you can.
Land softly in a squat position and, without pausing, jump to the side and repeat the movement pattern

2 Sets / 8 Reps / 2 min duration

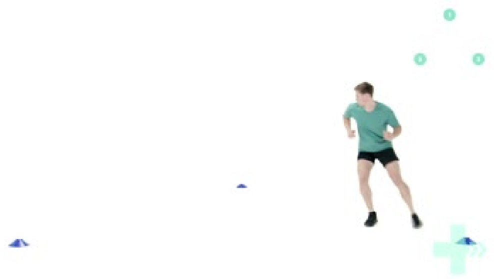

25. "COD (shuffle)" Fwd shuffle/sidestep/bwd shuffle, CCW/CW, cones in a triangle; 01

Place three cones on the ground in front of you, equidistant apart, to form a triangle shape, as shown in the diagram.
Start next to cone one facing between cone two and three.
When ready, travel forwards to cone two, then sidestep to cone three and then travel backwards to cone one.
Next, travel forwards to cone three, then sidestep to cone two and then travel backwards to cone one.
Repeat.
