# Supplementary material for: Novel motivational interviewing‐based intervention improves engagement in physical activity and readiness to change among adolescents with chronic pain
Source: Health Expect. 2024 Mar 31;27(2):e14031. doi: 10.1111/hex.14031 (PMC10982597; doi:10.1111/hex.14031)
Supplement: Supplementary file 7 — Appendix 2.5 Individual exercise program (A28). [file HEX-27-e14031-s009.pdf]

2 Sets / 8 Reps / 3 s hold

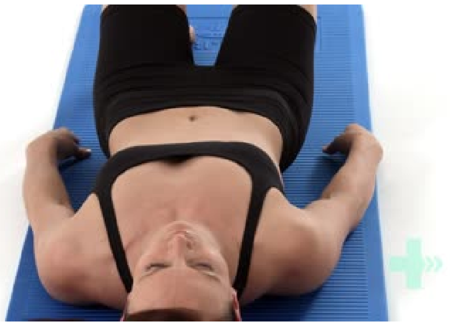

1. Core/pelvic floor strengthening isometric, supine

Lie on your back, with your leg bent and feet flat on the floor hip-width apart.
To activate your pelvic floor try to contract your anus, vagina and urethra inward and upward.
You can imagine that you are trying to pull a zipper closed with your pelvic floor from the bottom up.
Then try to draw your lower abdomen slightly down and in.
Hold this position.
Your breathing should remain steady throughout.
Relax and repeat.

2 Sets / 8 Reps / 20 sec duration

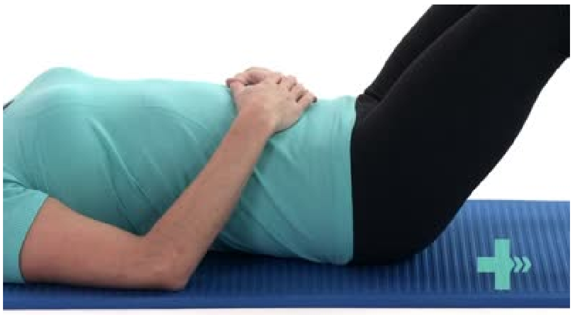

2. Breathing control, into nose/out through mouth - abdominal activation

Lie on your back with your knees bent and your feet flat on the floor.
Place your hands on the lower part of your stomach.
Take a deep breath in through your nose, taking the air right down into the base of your lungs.
You should feel your stomach push out against your hands.
Breathe out through your mouth, gently tensing the stomach muscles under your hands until your lungs are completely emptied.
You should feel your stomach push out into your hands as you inhale, and pull inwards as you exhale.
Repeat the deep inhaling and exhaling.

2 Sets / 8 Reps

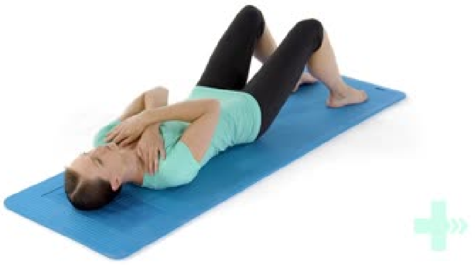

3. "Pelvic clock" Core mobility, supine, feet standing

Lie on your back with your legs bent and feet flat on the floor.
Your legs should be hips distance apart.
Imagine a clock face lying flat on the floor underneath your pelvis.
12 o'clock is closest to your head and 6 o'clock closest to your feet.
Tighten your buttock and abdominal muscles and imagine rocking your pelvis towards 12, then down towards 6.
Continue this motion, rocking between 12 and 6.
Now start to rock in diagonal motions towards 1 o'clock, then back to the center.
Rock to 2 o'clock, back to the center.
Rock sideways to 3 o'clock then back to the center.
Continue around the clock.

2 Sets / 8 Reps / 3 s hold

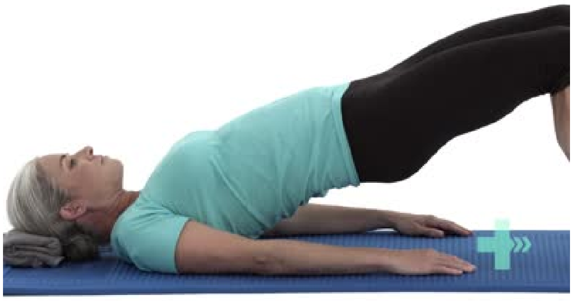

4. "Bridge" Core/gluteals strengthening; 01

Lie on your back with your knees bent and your feet flat on the floor.
Gently tilt your pelvis as if you are imprinting your lower back into the floor and lift your hips up into the air while still holding your pelvis level.
Hold in the bridge position before you then lower, keeping your navel drawn in and slowly lowering your spine back down onto the floor, one vertebrae at a time.
Keep your buttocks tight, until your pelvis rests back down on the floor.

2 Sets / 8 Reps

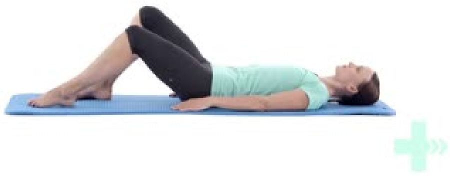

5. Core/pelvic floor stabilization, extending leg, slides, supine, feet standing

Lie on your back with your legs bent and feet flat on the floor.
Ensure your feet and knees are hip-width apart.
Tighten your abdominal and pelvic floor muscles, then slowly straighten one leg, sliding it along the floor.
Ensure you keep your body and hips level throughout this movement.
Control the movement as you slowly bring your leg back up to the starting position.
Repeat this movement with the same leg.

2 Sets / 8 Reps

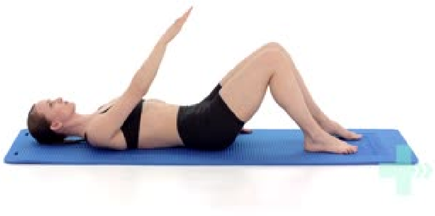

6. Core/pelvic floor stabilization, lifting arm overhead/extending opposite leg, supine, feet standing

Lie on your back with your knees bent and feet flat on the floor.
Ensure you maintain a hips distance between your feet and knees.
Tighten your pelvic floor and abdominal muscles, ensuring you keep a small arch in your lower back.
Keeping the tension in your core stability muscles, lift one arm forwards and up towards the ceiling.
Simultaneously slide your opposite foot along the floor until your leg is straight.
Continue the movement, raising your arm overhead.
Your lower and upper back should stay in the same position throughout this movement.
Once you have moved as far as you can go, control the movement as you simultaneously bring your arm back down and bend your leg back in.
Relax your core stability muscles before you repeat the sequence again.

2 Sets / 8 Reps

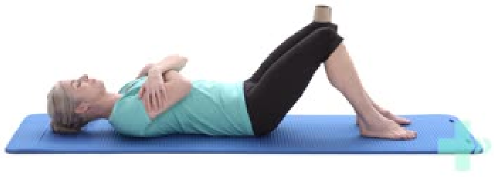

7. "Knee fall out" Core/abdominal stabilization, cup on opposite knee, supine

Lie on your back with your knees bent and your feet flat on the floor.
Place a plastic cup upside down on the knee of your weakest leg.
Tighten your core stability muscles before slowly dropping your stronger leg out to the side.
The weaker leg should remain still, making sure the cup does not fall as your strong leg moves.
Keep your core muscles working whilst you bring your knee back up to the starting position.
Do not allow your pelvis to rotate during this movement.

2 Sets / 8 Reps / 3 s hold

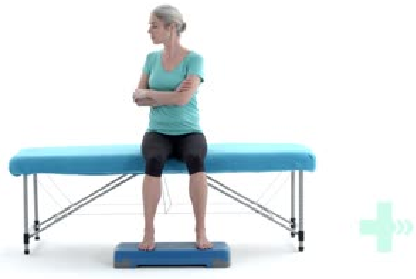

8. Upper trunk rotation AROM, arms crossed, sitting on table

Sit upright on a firm surface with your feet hip-width apart.
Sit far enough back so that your thighs are supported on the surface.
Fold your arms across your chest.
Rotate your body around to one side, turning your head with the movement.
Return to the center, and then repeat to the other side.

2 Sets / 8 Reps

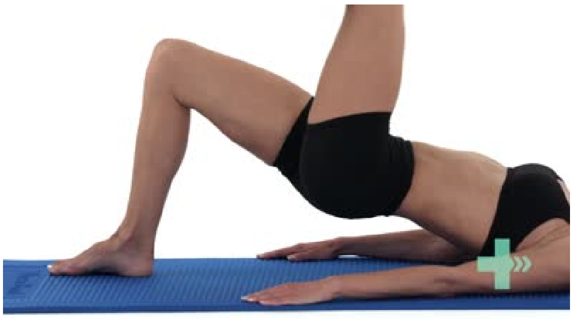

9. Pilates bridge level 1

Lie on your back with your legs bent and your feet flat on the floor.
Exhaling, lift your pelvis off the mat and move up into the Pelvic Curl position with your shoulders and arms pressing onto the mat.
This will be your starting position.
INHALE: raise one leg upwards, keeping your knee bent at 90 degrees, with the foot Plantar flexed (pointed).
EXHALE: lower your leg down and tap the floor with your toes.
INHALE: raise the same leg up, keeping a consistent angle at the knee.
EXHALE: lower your leg down, keeping your pelvis absolutely stable.
Continue on for the desired number of repetitions and repeat the movement with your other leg.
Keep your body stable as your leg hinges at the hip joint.
Maintain your pelvis at the same height throughout.

2 Sets / 8 Reps / 3 s hold

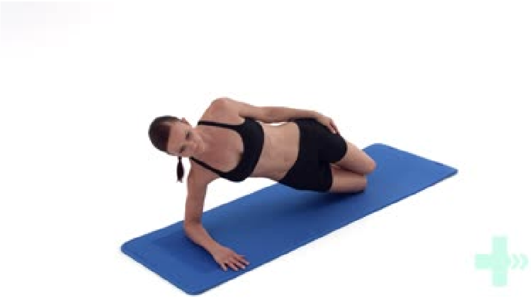

10. "Plank, side (low)" Core/scapular strengthening isometric, on knees

Lie on your side and prop yourself up on your elbow.
Bend your knees and lift your hips off the mat until you have a straight line from your knees to the top of your head.
Hold this position for as long as you can.

2 Sets / 8 Reps

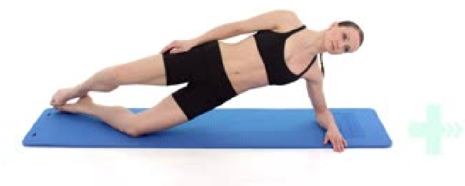

11. "Clamshell" Hip external rotation strengthening, in side plank

Lie on your side with your knees bent and feet stacked one on top of the other.
Prop yourself up on your forearm with your elbow under your shoulder.
Tighten your abdominal and buttock muscles, and then lift your hips up off the floor.
You should have a straight line from your head to your knees.
Holding this position, lift your top knee up, keeping your feet together.
Control the movement as you lower your knee back down again and repeat.

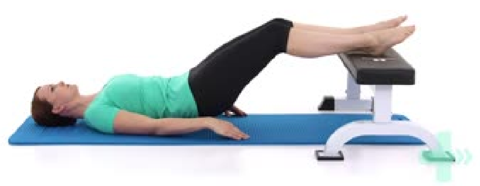

2 Sets / 8 Reps / 3 s hold

12. "Bridge" Core/hip stabilization, lifting leg, feet on bench

Lie on your back with your heels rested securely on a bench.
Ensure your knees and feet are hips distance apart with your knees pointing to the ceiling.
You should have your knees bent to approximately 45 degrees.
Tighten your abdominal and buttock muscles and roll your tail bone up from the floor.
Continue this movement, lifting your hips directly up to the ceiling until you have a straight line from your shoulders to your knees.
Keep your neck and shoulders relaxed.
Holding this position, lift one leg from the bench.
The leg remaining in contact with the bench will be the one you exercise.
Keep yous hips level throughout.
Lower your elevated back leg down to the bench.
Control the movement as you lower your hips back down to the floor.
Your abdominal muscles should remain engaged until your lower back reaches the floor.

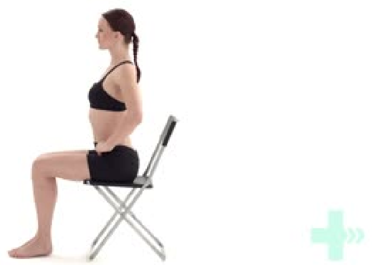

2 Sets / 8 Reps / 3 s hold

13. Posture, finding neutral spine, pelvic tilting, sitting; 01

Sit in a chair with your hands on your hips.
Round your shoulders and upper back, slouching to sit through your tail bone.
Roll on to your seat bones, opening up your chest and bringing your shoulders back and down.
Ease back a little.
This is good upright posture.

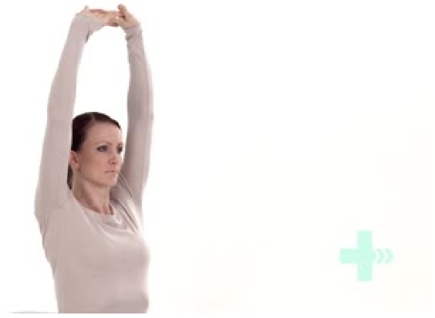

2 Sets / 8 Reps / 3 s hold

14. "Overhead stretch" Shoulder extensors, elbow/wrist flexors stretch, sitting; 02

Start in a seated position and interlock your fingers.
Raise your arms above your head and rotate your hands so they are facing palm up.
Push your arms upwards, feeling the stretch through your sides and shoulders.
Hold this position before you relax and repeat the movement again.

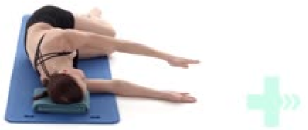

2 Sets / 8 Reps / 3 s hold

15. Upper trunk rotation AROM, leading with arm, legs 90/90, side lying

Lie on your side with your head supported on a pillow and your knees bent at 90 degrees.
Reach your arms out in front of you, with your hands together.
Rotate the upper arm upwards and outwards from your trunk.
Try to rotate as far as you can, without forcing the shoulder.
Hold in the maximum position.
Note: You can adjust the level at which you rotate by adjusting the angle of your knees.
