# Supplementary material for: Novel motivational interviewing‐based intervention improves engagement in physical activity and readiness to change among adolescents with chronic pain
Source: Health Expect. 2024 Mar 31;27(2):e14031. doi: 10.1111/hex.14031 (PMC10982597; doi:10.1111/hex.14031)
Supplement: Supplementary file 8 — Appendix 2.6 Individual exercise program (A32). [file HEX-27-e14031-s003.pdf]

2 Sets / 8 Reps

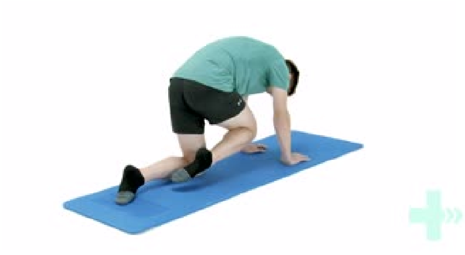

1. Neutral spine/scapular stabilization, quadruped, extending leg to flexing leg and trunk

Kneel down with your hands under your shoulders and knees under your hips.
Find the neutral position of your spine.
With your shoulders strong and your core activated, extend one leg back behind you until it is in line with the rest of your body.
Do not allow your back to dip.
Bring this leg back in, bending the knee in towards your chest.
Allow your back to round.
Repeat the movement, increasing the speed while maintaining good control of your body.

2 Sets / 8 Reps / 2 s hold

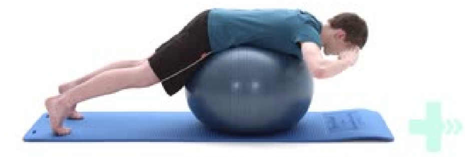

2. Trunk extension strengthening, prone on stability ball

Kneel in front of a stability ball.
Roll forwards over the ball until your pelvis rests on the ball.
Keep your feet on the floor at all times.
Place your hands near your ears.
Slowly lift your head, shoulders and chest off the ball, extending through your lower back.
Control the movement as you lower back down.

2 Sets / 8 Reps / 2 s hold

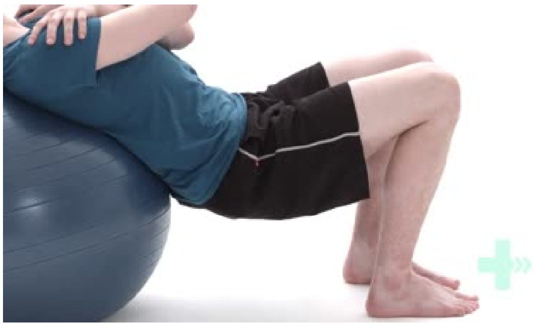

3. "Bridge" Core/gluteals strengthening, supine on stability ball; 01

Sit upright on stability ball.
Slowly walk your feet forwards allowing your back and then shoulders to rest on the ball.
Lower your buttocks down towards the floor.
Squeeze your buttocks and lift your hips up until you have a straight line from your chest to your knees, like a table.
Control the movement as you lower your hips back down.
Finish by walking your legs back in and sitting up on the ball.

2 Sets / 8 Reps

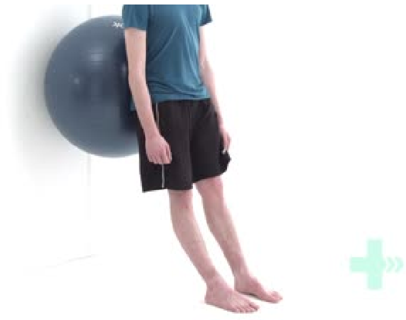

4. "Squat, stability ball on wall" Lower body strengthening; 01

Stand upright and place a stability ball in the small of your back.
Rest against a wall with your feet hip width apart.
Walk your feet forwards.
Bend your knees and use the ball to slide down the wall as far as you can comfortably go.
Your hips should drop directly down towards the floor.
Push back up, driving the movement through your buttock muscles.

2 Sets / 8 Reps

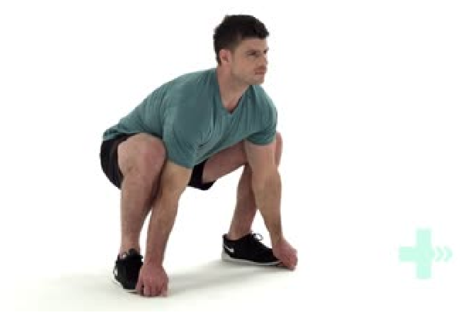

5. Hamstring stretch from squat, hands on floor

Stand tall with your feet wider than hip-width apart.
Bend forward at the waist to grab your toes with your hands.
Drop down into a deep squat while keeping your arms straight, elbows inside your knees, back flat, and chest up.
While holding your toes, raise your hips back and straighten your knees until you feel a good stretch in the back of your legs.
Reverse the movement pattern and return to the starting position.

2 Sets / 8 Reps / 1kg weight

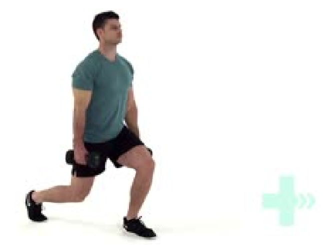

6. "Squat, split" Lower body strengthening, with dumbbells

Stand with your feet hip-width apart holding a dumbbell in each hand with your arms straight and by your side.
Take a long step forward into a staggered stance.
Squat down until your front thigh is parallel to the ground.
Keep your trunk upright throughout the movement with the head up and your gaze forward.
Keep your knees inline with your toes, toes pointing straight forward.
Then push back up and straighten your knees.

2 Sets / 8 Reps

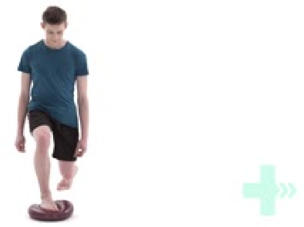

7. Balance, standing, lunging forward, onto wobble cushion

Stand up straight facing a wobble cushion.
Step your affected leg onto the cushion.
Lunge down, bending both knees and hips to 90 degrees.
Push through both feet to step your affected leg back to the starting position.
Ensure your knees travel forwards over your toes as you lunge down.

2 Sets / 8 Reps / 1 min duration

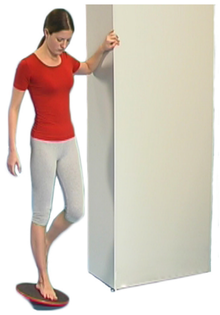

8. Standing ankle tilts (balance board) - version 1

Stand on one leg on a wobble board.

Rock the board first forwards and backwards, then side to side.

2 Sets / 8 Reps / 1 min duration

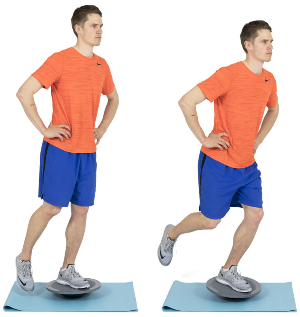

9. Single-leg Squat on a Balance Board

Stand on one leg on a balance board.

Squat down and push back up again while trying to keep the balance board level. Your hip, knee, ankle, and toes should stay aligned.

Note: Place the balance board on a surface that is not slippery.

2 Sets / 8 Reps / 1 min duration

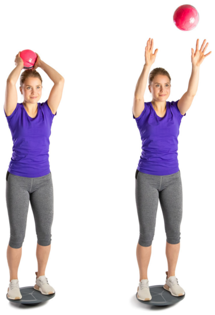

10. Balancing on a Balance Board – Throwing and Catching a Ball

Start by standing on a balance board.
Balance on the board and try to keep the edges off the floor.
Throw the ball from over your head towards a partner or against a wall and then catch it.

2 Sets / 8 Reps

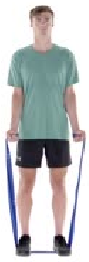

11. Elbow flexion biceps brachii strengthening, with band, 0 to 180° (bilateral), standing

Stand up straight with a resistance band placed firmly under your feet.
Hold one end of the tensioned band in each of your hands with your palms facing forwards.
Keeping your arms by your sides, slowly bend your elbows against the resistance of the band.
Control the movement as you lower your arms back down to the start position. Pause, and then repeat.

2 Sets / 8 Reps

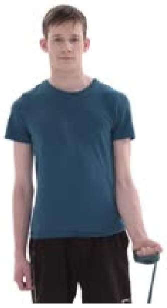

12. "Biceps curl" Elbow flexion strengthening, with band (low), standing

Stand up straight with one end of the resistance band firmly under your foot.
Hold the other end of the tensioned band in your affected hand.
Keeping your arm by your side, slowly bend your elbow, pulling against the resistance of the band.
Control the movement as you lower you arm back down.

10x2 mindkét karral, lassan!

2 Sets / 8 Reps

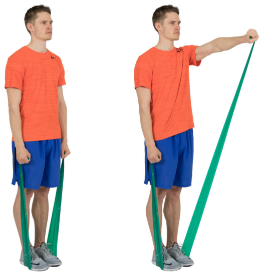

13. Alternating resisted shoulder flexion in standing - to shoulder level (palm facing down)

Stand tall with your trunk activated and knees slightly bent. Hold the ends of an exercise band that is placed under your feet.

Lift one arm forward while keeping it straight (palm facing down). Then, in a controlled manner, return to the starting position and repeat with the other arm.

Note:
- Do not shrug your shoulders.
- Keep your lower back in a neutral position (abdominals and buttocks tight).

2 Sets / 8 Reps / 2 s hold

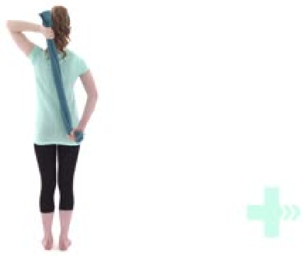

14. "Hand behind back" Shoulder internal rotation AAROM, with towel, standing; 01

Stand up straight.
Take a towel and drape it over your good shoulder.
Reach behind your back with your symptomatic arm and hold the other end of the towel.
With your top hand pull the towel straight up so that your back hand comes up towards your shoulder blade.
You will feel this stretch in the front of your shoulder.

2 Sets / 8 Reps

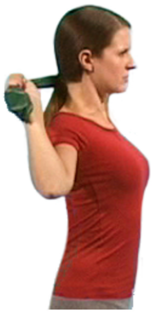

15. Resisted Shoulder Flexion/External Rotation in Standing

Stand or sit. Hold an exercise band in front of you with your hands shoulder-width apart and lift your arms.

Bring your hands down behind your head, pulling the band.

2 Sets / 8 Reps

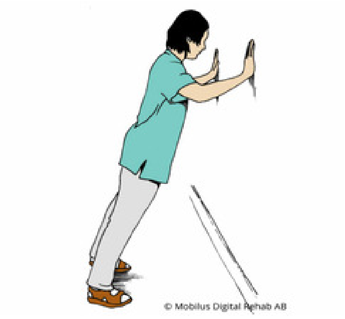

16. Wall Push up

Stand facing a wall, feet are shoulder-width apart and a couple of feet away from the wall. Tighten the abdomen and buttocks and keep your trunk in a straight line from head to foot. Place your hands against the wall in front of you and bend and straighten your elbows, keeping your trunk and head aligned.

2 Sets / 8 Reps

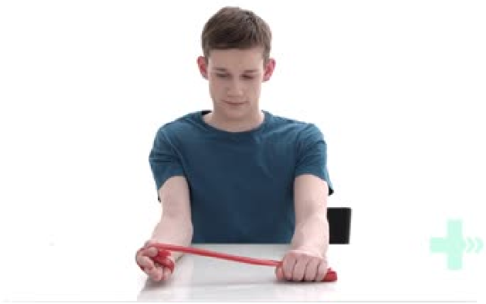

17. Forearm supination strengthening, with band; 05

Sit upright with your forearms resting on a table.
Hold a tensioned resistance band in both hands with your palms facing down.
Slowly turn the palm of your affected arm over, pulling against the resistance of the band.
Control the pull of the band as you return to the starting position.

2 Sets / 8 Reps

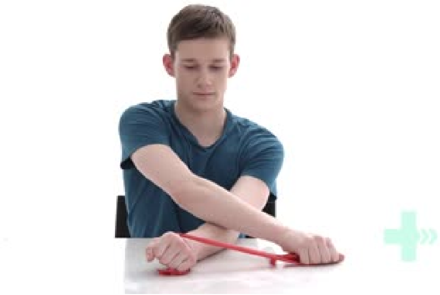

18. Forearm pronation strengthening, with band, arms crossed; 03

Sit upright on a chair with your forearms resting on a table.
Hold a tensioned resistance band in both hands.
Cross your good arm over the top of your affected arm.
Ensure the palm of your affected hand is face up.
Turn the palm of your affected arm over, pulling against the resistance of the band.
Control the pull of the band as you return to the starting position.

1 Set / 5 Reps / 2 s hold

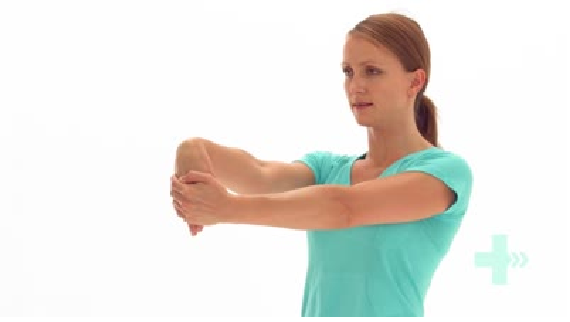

19. Wrist extensors stretch, palm down, standing

Extend your affected arm straight out in front of you with your palm face down, and drop your hand towards the floor.
With your other hand, apply a gentle pressure to the back of your wrist and hold.
You should feel this stretch down the back of your forearm.

Ezt az iskolában tudod csinálni, amikor megállsz az írással.

1 Set / 5 Reps / 2 s hold

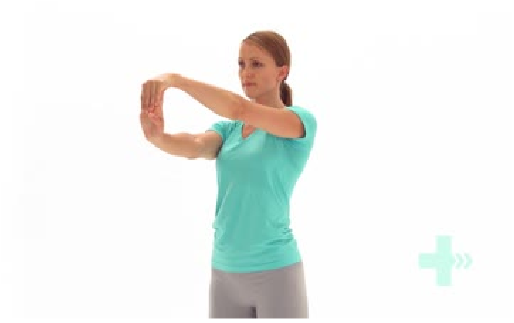

20. Wrist flexors stretch (alternate), arms forward, standing

Extend your arm straight in front of you and extend your wrist back towards your body.
With your other hand, apply gentle pressure and hold for a few seconds.
Repeat on the opposite side.

Ezt az iskolában tudod csinálni, amikor megállsz az írással.

1 Set / 5 Reps / 2 s hold

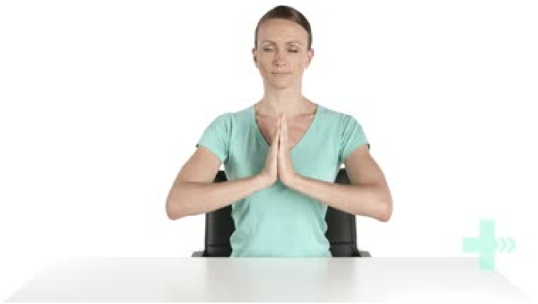

21. Wrist flexors stretch, palms together, sitting; 01

Sit upright in a chair.
Bring your palms together in front of you and close to your chest.
Gradually lower your hands, keeping the palms of your hands together.
Hold this position in a stretch.

Ezt az iskolában tudod csinálni, amikor megállsz az írással.

1 Set / 5 Reps / 2 s hold

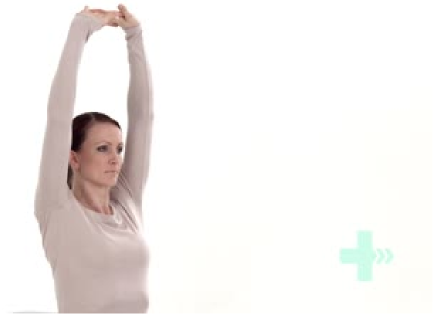

22. "Overhead stretch" Shoulder extensors, elbow/wrist flexors stretch, sitting; 02

Start in a seated position and interlock your fingers.
Raise your arms above your head and rotate your hands so they are facing palm up.
Push your arms upwards, feeling the stretch through your sides and shoulders.
Hold this position before you relax and repeat the movement again.

Ezt az iskolában tudod csinálni, amikor megállsz az írással.

1 Set / 1 Rep / 1 min duration

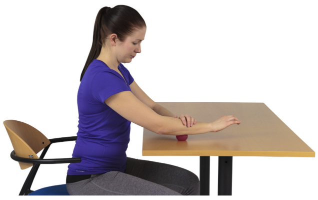

23. Seated wrist flexor and pronator self-myofascial release

Sit at a table with a ball under your forearm and palm facing downwards.

Add pressure with your other hand and start rolling the arm over the ball back and forth.

Ezt otthon vagy az iskolában tudod csinálni, amikor megállsz az írással.

1 Set / 1 Rep / 1 min duration

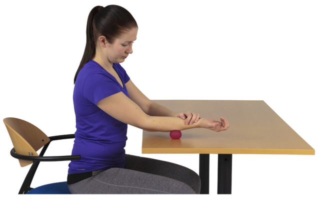

24. Seated wrist extensors and supinator self-myofascial release

Sit at a table with a ball under your forearm and palm facing upwards.

Add pressure with your other hand and start rolling the arm over the ball back and forth.

Ezt otthon vagy az iskolában tudod csinálni, amikor megállsz az írással.
