# Supplementary material for: Novel motivational interviewing‐based intervention improves engagement in physical activity and readiness to change among adolescents with chronic pain
Source: Health Expect. 2024 Mar 31;27(2):e14031. doi: 10.1111/hex.14031 (PMC10982597; doi:10.1111/hex.14031)
Supplement: Supplementary file 9 — Appendix 2.7 Individual exercise program (A33). [file HEX-27-e14031-s001.pdf]

2 Sets / 8 Reps

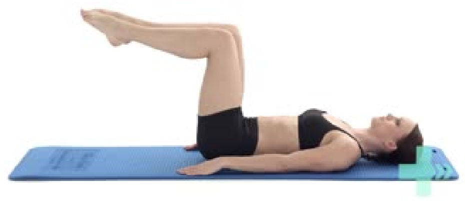

1. Core/abdominal stabilization, extending leg (alternate), legs 90/90, supine

Lie on your back and bring your legs up to a table-top position with your hips and knees at a right angle.
Relax your upper body as you extend one leg down to the floor, whilst maintaining the table-top position with the other.
Bring this leg back up and repeat with the other leg.
Ensure you keep your core strong and back flattened to the floor throughout this exercise.

2 Sets / 8 Reps / 2 s hold

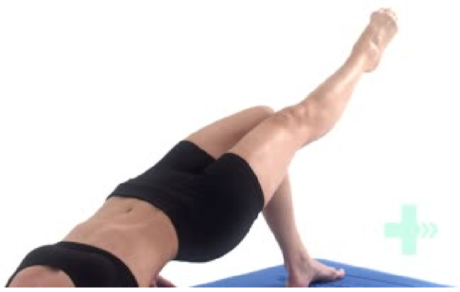

2. "Bridge" Core/hip stabilization, lifting leg to ceiling

Lie on your back with your knees bent and your feet flat on the floor.
Tighten your buttock muscles and lift your hips up into the bridge position.
Lift one foot off the floor then straighten your knee until it is aligned with your other thigh.
Hold this position and lift your elevated leg up and down, keeping it straight.
Make sure you keep your hips up and level throughout the movement.

2 Sets / 8 Reps / 2 s hold

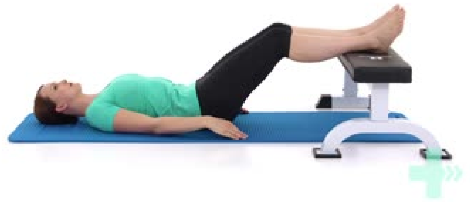

3. "Bridge" Core/gluteals strengthening, feet on bench

Lie on your back with your heels rested securely on a bench.
Ensure your knees and feet are hips distance apart with your knees pointing to the ceiling.
You should have your knees bent to approximately 45 degrees.
Tighten your abdominal and buttock muscles and roll your tail bone up from the floor.
Continue this movement, lifting your hips directly up to the ceiling until you have a straight line from your shoulders to your knees.
Keep your neck and shoulders relaxed.
Control the movement as you lower your hips back down to the floor.
Your abdominal muscles should remain engaged until your lower back reaches the floor.

2 Sets / 8 Reps

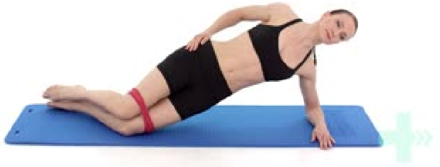

4. "Clamshell" Hip external rotation strengthening, with band, in side plank

Lie on your side with your knees bent and feet stacked one on top of the other.
Tie a resistance band around your thighs, just above your knees.
Prop yourself up on your forearm with your elbow under your shoulder.
Tighten your abdominal and buttock muscles, and then lift your hips up off the floor.
You should have a straight line from your head to your knees.
Holding this position, lift your top knee up, keeping your feet together.
Control the movement as you lower your knee back down again and repeat.

2 Sets / 8 Reps / 2 s hold

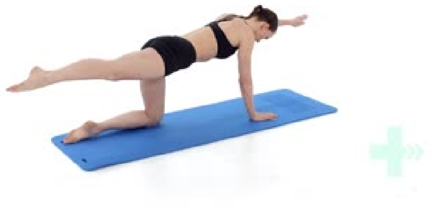

5. "Bird dog" Core/pelvic floor stabilization, with hip/trunk flexion, LBP

Start on your hands and knees, with your hands under your shoulders, and knees under your hips.
Tighten the abdominal and pelvic floor muscles.
Straighten your opposite leg and opposite arm simultaneously, making sure your maintain good control in your torso.
Do not allow your body or hips to rotate.
Hold this position, then bring both the elevated arm and leg in towards your belly button, aiming to touch your knee to your elbow.
Allow your back to bend to do this.
Extend your arm and leg again to repeat the movement.

2 Sets / 8 Reps / 2 s hold

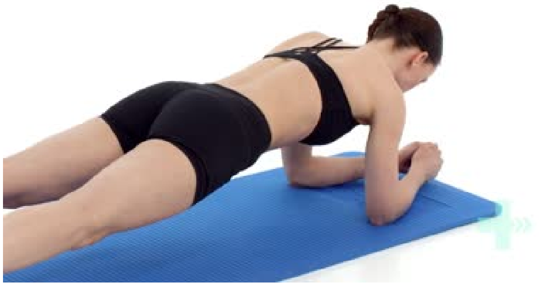

6. "Plank (low)" Core/scapular strengthening isometric, how to

Lie on your front propped up onto your forearms.
Tuck your toes under with your legs hips width apart.
Tighten your abdominal and buttock muscles, then slowly lift your body and hips up so that your weight is on your knees.
Next, lift your knees from the floor until you only have your forearms and toes in contact with the ground.
Drive your elbows into the ground to strengthen your shoulders while maintaining your gaze between your hands.
You should have a straight line from the top of your head to your heels.
Ensure your back does not sag.

2 Sets / 8 Reps

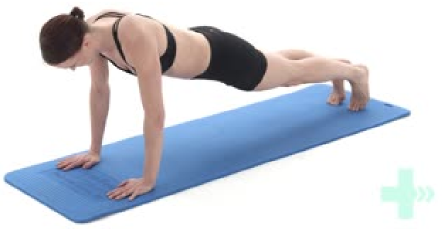

7. "Push up plus" Chest/shoulder strengthening into scapular protraction

Lie on your front with your hands just wider than your shoulders and toes on the floor.
Tighten your abdominal and buttock muscles as you push yourself up through your arms, keeping your body straight.
You should have a straight line from your shoulders to your heels.
Drive the heels of your hands into the floor so that you fix your shoulder blades fully against your rib cage.
Be careful not to just round your upper back.
Keeping this control at the shoulder blades, bend your elbows, dropping down into a press-up.
Push back through the heels of your hands to straighten your elbows out again.
Ensure you do not allow your hips or abdomen to sag.
Repeat this movement.

2 Sets / 8 Reps

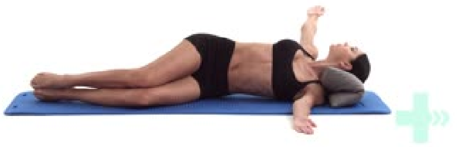

8. Pilates arm opening

Lie on your side with your head resting on a small cushion.
Bend both legs at a 45 degree angle to the hips, keeping them together and straighten your arms out in front of your body with one arm on top of the other.
INHALE: raise your top arm up towards the ceiling, followed by your head and upper body
EXHALE: continue rotating the spine, as you lower your straight arm further down
INHALE: bring your arm back, reaching to the ceiling
EXHALE: lower your arm down to the starting position
Allow your head to follow the movement of your arm.
Hold the stretch and engage your abdominals as you bring your arm back over and down to the starting position.

2 Sets / 8 Reps / 2 s hold

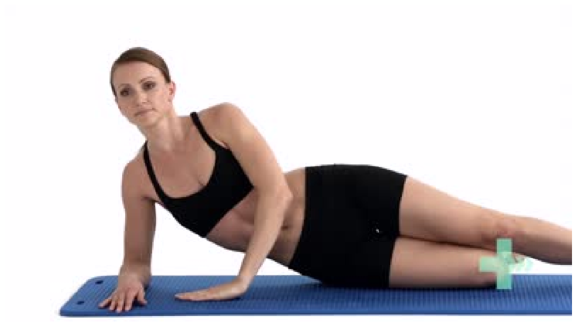

9. "Plank, side (low)" Core/scapular strengthening isometric, arm to ceiling

Lie on your side and prop yourself up on your elbow.
Your bottom leg is bent for support and your other leg is straight.
Lift your hips off the floor so that your body is in a straight line.
Reach up with your upper arm and hold this position for a moment.
A more advanced version of this exercise can be performed with both legs straight.

2 Sets / 8 Reps / 2 s hold

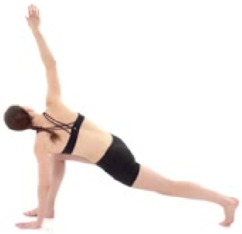

10. Iliopsoas stretch, with trunk rotation, deep split stance; 02

Stand up straight and take a large step forwards onto one leg.
Bend this front leg and sink your hips down.
Place your hands on the floor on the inside of your front foot.
Your back leg should remain straight, balanced on the ball of your foot.
Your front knee should not travel further forwards than your toes.
You may need to shuffle your foot forward to achieve this.
Keeping your hips pushed towards your front foot, lift the your arm on the same side as your front foot up towards the ceiling.
Allow your head and body to turn and follow this movement.
Hold this position before returning your hand to the floor.

2 Sets / 8 Reps / 2kg weight

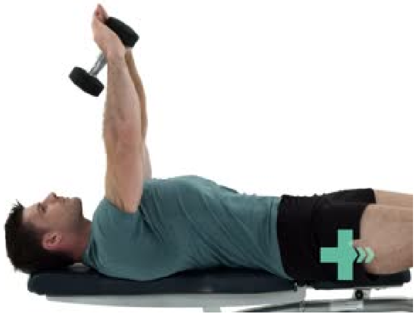

11. "Latissimus pullover" Core/shoulder extension strengthening, with dumbbell

Lie on a bench holding a dumbbell directly overhead with your arms fully extended, and your abdominals tensed.
Lower your arms back down over your head without bending your elbows, and then return to the starting position.
Continue for the required number of repetitions in a fluid, controlled manner.

2 Sets / 8 Reps

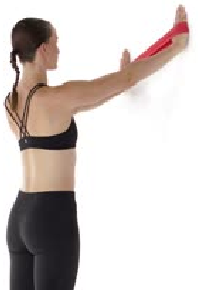

12. Scapular wall slide series - 1) Elevation/upward rotation 2) Retraction 3) Depression/downward rotation, with band

Stand up straight with a resistance band looped around both hands.
Facing a wall, place the palms of your hands on the wall around shoulder height.
Your hands should be around shoulder distance apart with tension in the band.
Drive the heels of your hands into the wall so that your shoulder blades hug tightly to your rib cage.
Keep your neck long.
Fixing one hand in position, slide the palm of your other hand diagonally up and out.
Return to the starting position, and slide your palm directly out to the side.
Return to the starting position, and finally slide your diagonally down and out.
Return to the starting position and repeat.
Your palms should remain in contact with the wall throughout, with your arms straight and your abdominal muscles engaged.

2 Sets / 8 Reps / 2 s hold

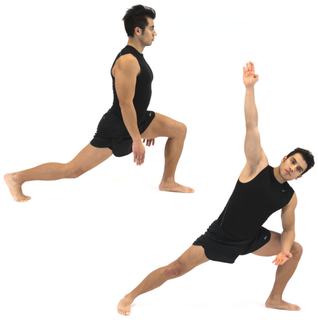

13. Warrior Angle Pose

Lunge position.
Lengthen the spine.
Turn, lean your elbow against your thigh and reach the other arm towards the ceiling.

2 Sets / 8 Reps

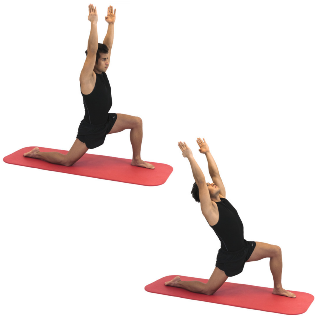

14. Psoas Opening

Kneel on one knee with the other leg out in front.
Lift your arms up.
Straighten your hip and tighten your lower abdominals.
Breathe in and lengthen the spine. Breathe out and reach up.
Open your chest and bend backwards.
Hold and breathe deeply.

2 Sets / 8 Reps / 2 s hold

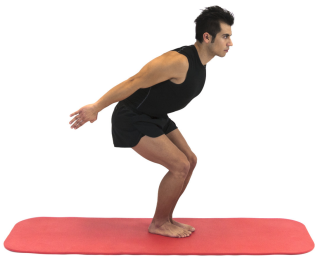

15. Power Pose - Version 2

Stand tall with feet together. Breathe in.

Breathe out, sit down and pushing the sitting bones back. Extend your arms. Keep the chest open. Hold as long as is directed.

2 Sets / 8 Reps / 2kg weight

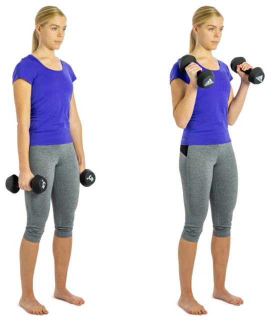

16. Hammer Curl with Dumbbells

Stand tall with your arms by your side, holding dumbbells in both hands. Palms are facing inwards.

Bend your elbows and bring your thumbs towards your shoulders. Return to the starting position in a controlled manner.

2 Sets / 8 Reps / 2kg weight

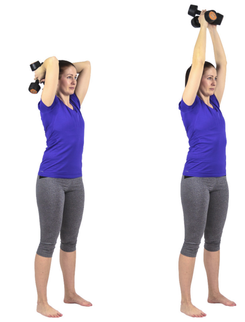

17. Overhead Triceps Extension

Stand tall holding weights above your head, elbows bent and pointing upwards.

Straighten your elbows and bend back to the starting position in a controlled manner.

Note:
- When bending/straightening your elbows try not to move your upper arms and don't let your elbows open to the side.
- Draw in the abdominals to control the position of your lower back.

2 Sets / 8 Reps / 2kg weight

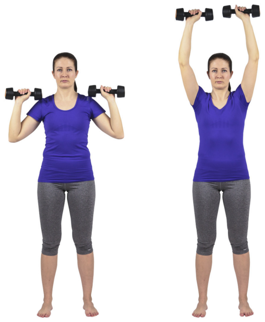

18. Resisted Shoulder Press

Stand tall. Hold weights at shoulder height with your elbows pointing to the sides.

Press the weights up to straight arms. Return to the starting position in a controlled manner.

Note:
- Try not to shrug your shoulders.
- Keep abdominals tight to avoid excessively extending your lower back.

2 Sets / 8 Reps / 2kg weight

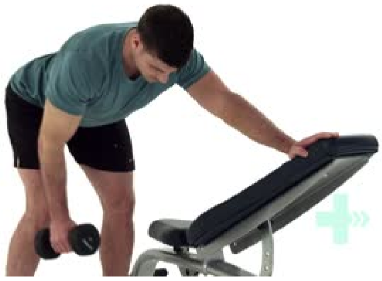

19. "Row, bent over" Core/scapular stabilization, with dumbbell

Stand hinged over at the waist holding a dumbbell in one hand.
Keeping your back aligned, slide your shoulder blades back and down towards your backside.
Pull the dumbbell upwards towards the side of your rib cage.
Lower the weights back to the starting position.
Complete the set on one side before repeating with the opposite arm.
If required use a waist high object to provide support with your other hand.

2 Sets / 8 Reps

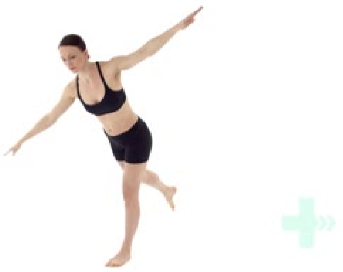

20. "Hip hinge, single leg" Posterior chain strengthening, windmill arms

Stand up straight and balance on one leg.
Lift your arms to the side and look straight ahead.
Keeping your back straight, lean forwards, pushing your hips back behind you until your back is horizontal.
You may need to soften your knee a little.
Lift your elevated leg directly back behind you.
Keeping your back and elevated leg straight, bring one hand down and across to reach the opposite side of your stance foot.
Your other arm should turn up to point at the ceiling.
Allow your body to rotate with the movement and your head to turn to look at your upper hand.
Come back to the centre, and rotate to the other side.
Continue this movement.

2 Sets / 8 Reps / 2kg weight

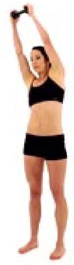

21. "Woodchop" Trunk rotation/flexion strengthening, with weight

Hold a weight in both hands and stand up straight.
Lift the weight high up to one side, then bring it down across the body, to the opposite hip.
Imagine you are chopping wood with an ax.
Make sure you keep your back straight throughout this movement.
