# Supplementary material for: Novel motivational interviewing‐based intervention improves engagement in physical activity and readiness to change among adolescents with chronic pain
Source: Health Expect. 2024 Mar 31;27(2):e14031. doi: 10.1111/hex.14031 (PMC10982597; doi:10.1111/hex.14031)
Supplement: Supplementary file 10 — Appendix 3. Progressive muscle relaxation and breathing exercise scripts. [file HEX-27-e14031-s006.docx]

**Appendix 3**

**Progressive muscle relaxation**

Pay attention to your breathing, as you did during the previous breathing exercises. Breathe in deeply through your nose. Then, let the air out through your mouth. Slow down your breathing. Inhale evenly and let the air out evenly. Start the relaxation with your face. Your eyes should be closed.

Squeeze your eyelids together as if you were squinting. Then, let them go. Relax. Now, look surprised. Raise your eyebrows. Then, let them back down. Feel your face relax.

Now, bring your attention to your chin. Notice if your teeth are clenched or if your jaw is tight. Try to relax them. You can also open your mouth slightly. Breathe slowly. Evenly.

Now, think about your shoulders. Try to push them down towards your hips. Then bring them back. Now, pull your shoulders up to your ears, then release. Feel everything relax around your shoulders.

Now, think about your elbows. Stretch your arms out. Not too hard. Then relax your elbows. Now move down and watch your palms. Extend your fingers. Then relax them.

Take a deep breath through your nose. Now, let it out through your mouth.

Now bring your attention to your hips, squeeze your glutes, hold them, then relax them. Now, bring your attention to your knees. Tuck your knees into the ground, or if you are sitting, extend your knees. Then release them. Relax them.

Now move even lower onto your ankles, pulling your toes back towards your nose. He felt your calves tighten in the back. Then let go. Relax your ankles. Relax your toes.

Now, bring your attention back to your breathing. Breathe evenly. Long inhale. Long exhale.

Now start to look at your whole body. From the top of your head down to your toes.

Do you feel tightness anywhere? If so, try to relax that part of your body. Make every part of your body as loose as possible. Now, bring your attention back to your breathing.

Slowly let the air flow in. Slowly, it flows out. Repeat a few more times. Feel everything relax.

On your next exhalation, slowly open your eyes and come back into the room.

**Breathing exercise**

Let's start the breathing exercises. Settle into a comfortable position. This can be sitting, resting your head, or lying on your back. If you feel like it, put your hands on your stomach or, if you are sitting, on your armrest. You may want to put them in your lap or next to you. Wherever feels comfortable.

Now watch your feet. If they are crossed, spread them apart and just relax your knees. Think about your shoulders. And relax them too. Now, focus your attention on your breathing.

Breathe in slowly through your nose. Then, breathe out through your mouth. See if you can deepen your breathing. Start counting how many seconds it takes you to inhale and how many seconds it takes you to exhale. Now, try to equate it to the same amount of time for the air to flow in, and the same amount of time for it to flow out.

The air flows in. And it flows out.

Notice if your belly is loose when you breathe. Place your palm on your chest. And the other on your belly. See which palm rises more. The one on your chest or the one on your stomach.

Try to control your breathing so that the hand on your stomach rises more. Keep your breathing slow and steady. Let the air flow in through your nose. Then, it flows out through your mouth.

Notice how loose your palms are. If they feel tight, relax them. Then, bring your attention back to your breathing. Now, notice your shoulders if they feel tight. Relax them on the exhale. Keep breathing. Slowly. Evenly.

Feel everything relax. Repeat the breaths. Then, when you feel good, open your eyes to exhale and return to the room.
